# HLA-DQB1 6672G>C (rs113332494) is associated with clozapine-induced neutropenia and agranulocytosis in individuals of European ancestry

**DOI:** 10.1038/s41398-021-01322-w

**Published:** 2021-04-12

**Authors:** Bettina Konte, James T. R. Walters, Dan Rujescu, Sophie E. Legge, Antonio F. Pardiñas, Dan Cohen, Munir Pirmohamed, Jari Tiihonen, Annette M. Hartmann, Jan P. Bogers, Jan van der Weide, Karen van der Weide, Anu Putkonen, Eila Repo-Tiihonen, Tero Hallikainen, Ed Silva, Oddur Ingimarsson, Engilbert Sigurdsson, James L. Kennedy, Patrick F. Sullivan, Marcella Rietschel, Gerome Breen, Hreinn Stefansson, Kari Stefansson, David A. Collier, Michael C. O’Donovan, Ina Giegling

**Affiliations:** 1 Department of Psychiatry, Psychotherapy and Psychosomatics, Martin-Luther-University Halle-Wittenberg, Halle, Germany; 2 MRC Centre for Neuropsychiatric Genetics and Genomics, Division of Psychological Medicine and Clinical Neurosciences, School of Medicine, Cardiff University, Cardiff, UK; 3 Department of Severe Mental Illness, Mental Health Care Organization North-Holland North, Heerhugowaard, The Netherlands; 4 Department of Pharmacology and Therapeutics, The University of Liverpool, Liverpool, England; 5 Department of Forensic Psychiatry, University of Eastern Finland, Niuvanniemi Hospital, Kuopio, Finland; 6 Department of Clinical Neuroscience, Karolinska Institutet, and Center for Psychiatry Research, Stockholm City Council, Stockholm, Sweden; 7 Mental Health Services Rivierduinen, Oegstgeest, The Netherlands; 8 Department of Clinical Chemistry, St Jansdal Hospital, Harderwijk, The Netherlands; 9 Psychiatric Hospital GGz Centraal, Dependance Meerkanten, Ermelo, The Netherlands; 10 Faculty of Medicine, School of Health Sciences, University of Iceland, Reykjavík, Iceland; 11 Landspitali University Hospital, Mental Health Services, Hringbraut, 101 Reykjavik, Iceland; 12 Campbell Family Mental Health Research Institute, Centre for Addiction and Mental Health, Toronto, ON M5T 1R8 Canada; 13 Department of Psychiatry, University of Toronto, Toronto, ON M5T 1R8 Canada; 14 Institute of Medical Science, University of Toronto, Toronto, ON M5S 1A8 Canada; 15 Department of Medical Epidemiology and Biostatistics, Karolinska Institutet, Stockholm, SE-17177 Sweden; 16 Department of Genetics, University of North Carolina, Chapel Hill, NC 27599-7264 USA; 17 Department of Psychiatry, University of North Carolina, Chapel Hill, NC 27599-7160 USA; 18 Central Institute of Mental Health, Medical Faculty Mannheim, Heidelberg University, Mannheim, Germany; 19 King’s College London, London, UK; 20 deCODE Genetics/Amgen, Reykjavik, Iceland; 21 SGDP Centre, Institute of Psychiatry, Psychology & Neuroscience, King’s College London, London, UK; 22 Eli Lilly and Company, Bracknell, UK

**Keywords:** Clinical genetics, Predictive markers, Psychiatric disorders

## Abstract

The atypical antipsychotic clozapine is the only effective medication for treatment-resistant schizophrenia. However, it can also induce serious adverse drug reactions, including agranulocytosis and neutropenia. The mechanism by which it does so is largely unknown, but there is evidence for contributing genetic factors. Several studies identified *HLA-DQB1* variants and especially a polymorphism located in *HLA-DQB1* (6672G>C, rs113332494) as associated with clozapine-induced agranulocytosis and neutropenia. We analysed the risk allele distribution of SNP rs113332494 in a sample of 1396 controls and 178 neutropenia cases of which 60 developed agranulocytosis. Absolute neutrophil counts of 500/mm^3^ and 1500/mm^3^ were used for defining agranulocytosis and neutropenia cases, respectively. We also performed association analyses and analysed local ancestry patterns in individuals of European ancestry, seeking replication and extension of earlier findings. *HLA-DQB1* (6672G>C, rs113332494) was associated with neutropenia (OR = 6.20, *P* = 2.20E−06) and agranulocytosis (OR = 10.49, *P* = 1.83E−06) in individuals of European ancestry. The association signal strengthened after including local ancestry estimates (neutropenia: OR = 10.38, *P* = 6.05E−08; agranulocytosis: OR = 16.31, *P* = 1.39E−06), with effect sizes being considerably larger for agranulocytosis. Using local ancestry estimates for prediction, the sensitivity of rs113332494 increased from 11.28 to 55.64% for neutropenia and from 16.67 to 53.70% for agranulocytosis. Our study further strengthens the evidence implicating *HLA-DQB1* in agranulocytosis and neutropenia, suggesting components of the immune system as contributing to this serious adverse drug reaction. Using local ancestry estimates might help in identifying risk variants and improve prediction of haematological adverse effects.

## Introduction

Clozapine is the most effective antipsychotic medication, particularly for treatment resistant patients where clozapine is the only evidence based option^1–3^. Compared to alternative antipsychotics it offers superior symptom control, longer duration of treatment, reduced hospitalisation, improved cognition, better work and social function, a higher quality of life and reductions in violence and in suicidality^4,5^. However, due to serious adverse drug reactions, including agranulocytosis, clozapine treatment is only indicated in patients who have failed at least two other antipsychotics. To minimize the risk of agranulocytosis, frequent blood monitoring is required which represents another restriction to its use in an often challenging population of patients^6^.

The ability of clozapine to induce abnormally low concentration of neutrophils, which can predispose patients to serious and potentially lethal infections, has been recognised for several decades. Neutropenia and agranulocytosis describe absolute neutrophil counts (ANCs) below 1500 and 500 cells/mm^3^, respectively. In 1975, treatment of 2260 patients with clozapine was associated with agranulocytosis in 16 patients, 8 of whom died^7^, leading to clozapine withdrawal from the market in many countries. Clozapine was still prescribed in German speaking countries and Finland and reintroduced in other countries in 1990 under strict conditions which required regular blood count monitoring^8^. The incidence of neutropenia and agranulocytosis has recently been reported as 3.8% (95% CI: 2.7–5.2%) and 0.9% (95% CI: 0.7–1.1%), respectively^9^.

The mechanisms underlying clozapine-induced haematological adverse effects are largely unknown. There is evidence for toxic, immunological and genetic mechanisms indicating a multi-factorial pathogenesis. Agranulocytosis seems to be an idiosyncratic reaction, is dose-independent and is influenced by genetic or environmental factors^10^. Whereas neutropenia has been proposed to be a direct toxic effect of the parent drug, agranulocytosis seems to be the result of a toxic event involving an unstable intermediate metabolite, a nitrenium ion^10,11^. Binding of this ion to neutrophil proteins could lead to either a disturbance of neutrophil function or hapten formation, thereby triggering the immune destruction of the neutrophil^11^. The possibility of an underlying immunological mechanism is indicated by findings that a more severe and rapid course of agranulocytosis can occur after re-exposure to clozapine as well as studies identifying risk variants located in the major histocompatibility complex (MHC)^12,13^. Benign ethnic neutropenia (BEN) is characterized by low neutrophil counts not associated with an increased risk of infections and shows an increased prevalence in individuals of African or Middle Eastern ancestry, potentially due to the contribution of genetic variants that are rare elsewhere^14,15^. These ancestry dependent differences, and the fact that other adverse drug reactions also have genetic mediators, present a strong argument that genetic factors explain, at least partly, inter-individual variability and differences in bioactivation, detoxification and immunological response^11,16,17^.

Very few genetic markers have been clearly associated with clozapine induced agranulocytosis or neutropenia. Identified genetic risk factors include specific human leucocyte antigen (HLA) variants and haplotypes^12,18^. The HLA region was first implicated in small studies that identified the HLA allele DQB1*05:02 as risk factor for agranulocytosis in patients of European ancestry^19,20^. Evidence strengthened when Athanasiou et al. analysed 74 candidate genes in a cohort recruited from sites within the USA, Russia and South Africa in order to replicate the findings in a second cohort of non-Jewish schizophrenia patients of German descent^21^. Only variants in *HLA-DQB1* were significantly associated with agranulocytosis in both cohorts. A subsequent refinement analysis revealed association to a single SNP (*HLA-DQB1* 6672G>C, rs113332494, OR = 16.9), which is in strong linkage disequilibrium (LD) with DQB1*05:02^22^. Although two other smaller studies were not able to replicate these findings^23,24^, substantial evidence for the importance of the *HLA-DQB1* region has also come from the largest genome-wide association studies (GWAS) conducted to date^22,25^. The Clozapine-Induced Agranulocytosis Consortium (CIAC) analysed imputed HLA alleles and amino acid changes in 162 patients and 4319 controls. Clozapine treated cases (ANC ≤ 1000) were compared to two populations: patients with schizophrenia treated with clozapine for at least 1 year without developing neutropenia (ANC > 1500) and population controls. Two genome-wide associated variants were identified, including a single non-synonymous variant in *HLA-DQB1* (126Q) (OR = 0.19, *P* = 4.7E−14), for which the most common HLA allele at high-risk is HLA-DQB1*05:02^22^. Moreover, rs113332494 was genotyped in a British sample of 60 clozapine-treated cases and 305 clozapine-treated controls and again, significantly associated with neutropenia (OR = 15.6, *P* = 0.015). The association strengthened (OR = 38.1, *P* = 0.0079) using a stricter ANC threshold of 1000 mm^−3^ ^25^.

The variant rs113332494 is located in the MHC region on chromosome 6, a region with extensive LD spanning more than 3 Mb. The region is highly polymorphic and this variability is maintained by positive natural selection^26^. Population-specific recombination sites might contribute to the high diversity of haplotypes^27^ and it has been shown that the region exhibits an excess of local ancestry switches in non-European populations^26,28^. A local ancestry switch is defined as a genomic region exhibiting a change in ancestry, such that while most haplotypes across the broader region originate from one ancestral population, local haplotypes in a subregion might be derived from a different population as a result of recombination. Interestingly, an increase in East Asian ancestry haplotypes in the *HLA-DQB1* region was found in a neutropenia case–control sample of British ancestry^25^. The switch was seen in cases and controls, but nevertheless, local ancestry switches could be additional relevant factors influencing disease risk.

In order to replicate and refine previous findings and clarify the influence of the marker on the risk of neutropenia and agranulocytosis, we analysed the risk allele distribution, global and local population structure and association of the marker rs113332494 with neutropenia and agranulocytosis in 1396 controls and 178 neutropenia cases of which 60 developed agranulocytosis. To the best of our knowledge, this is the first study which determines the predictive power of rs113332494 incorporating local ancestry information.

## Methods

### Sample description

The CRESTAR sample comprised 1576 clozapine treated individuals, 1396 controls and 180 neutropenia cases of which 61 developed agranulocytosis (Supplementary Tables S1 and S2). Any psychiatric diagnosis was allowed; the primary psychiatric diagnosis was schizophrenia. ANC thresholds of 500/mm^3^ or 1500/mm^3^ were used for defining agranulocytosis and neutropenia cases, respectively. A small number of cases (*N* = 22, 3 agranulocytosis and 19 neutropenia cases) were classified by clinical judgement due to a precipitous drop in ANC while being intensively monitored. A detailed description of all contributing groups and inclusion criteria can be found in Supplementary Material.

### Overlap to other studies

Overlaps to CIAC^22^ and CLOZUK^25^ were examined by identity-by-descent estimations. Using genome-wide data we identified a small number of individuals (eight controls, seven cases) of European ancestry as duplicates in CIAC. These individuals were not excluded, as the marker, which is the focus of the present study, was not imputed at adequate quality in the GWAS conducted by that consortium. Overlap to CLOZUK cases was checked by identifying duplicates and first-degree relatives using ~1000 markers. One neutropenia and one agranulocytosis case were excluded from association and prediction analyses as they were found to be part of the CLOZUK study. See Supplementary Material for details and a sensitivity analysis excluding the individuals overlapping with CIAC.

### Genotyping/Imputation

Individuals were genotyped on Illumina HumanOmniExpress-12v1 and standard quality checks were applied (see Supplementary Methods). Pre-phasing was performed with SHAPEIT2^29^ and imputation with IMPUTE2^30,31^. We used the reference panel 1000 Genomes Project Phase 1 (August 2012), provided by the SHAPEIT2 authors, for both steps.

### Population structure

Population structure was analyzed by EIGENSTRAT (Supplementary Fig. S1) and an admixture analysis in a supervised setting using African (AFR), East Asian (EAS) and European (EUR) 1000 Genomes Project (1 KG) super populations as reference using ADMIXTURE v1.3.0^32^. African (*N* = 48) and European (*N* = 1006) subsamples were defined as individuals with at least 80% AFR or EUR ancestry fractions. All other individuals were defined as admixed (*N* = 522).

### Statistical analysis

The statistical analysis included 1574 individuals (1396 controls, 178 neutropenia cases of whom 60 developed agranulocytosis) once the two duplicate individuals from CLOZUK were excluded (see above).

Risk allele distribution of marker rs113332494 was examined based on best guess genotypes derived from PLINK v1.9^33^^,^^34^ using the following parameters: --hard-call-threshold 0.3, --geno 0.1, --maf 0.01, --hwe 1E-05. Genotypes with posterior probabilities below 0.7 were set to missing.

Association analysis was performed on individuals of European ancestry using logistic regression in PLINK v1.9^33,34^ applying an additive model corrected for relevant principal components inferred by EIGENSTRAT v6.0.1^35^. Supplementary Fig. S2 shows the scatterplot of first two principal components.

The main association analyses were conducted on neutropenia (ANC ≤ 1500) and agranulocytosis (ANC ≤ 500). To investigate the contribution of agranulocytosis cases, we excluded all cases with ANC ≥ 500 and tested a range of ANC thresholds (500–1500 in steps of 100). We also analysed different ANC thresholds excluding neutropenia cases for a range of ANC thresholds in steps of 100 to determine how rs113332494 behaves as a predictor.

Estimating local ancestry haplotypes across the extended MHC region (25–35 Mb) was performed with ELAI^36^ using the 1KG super populations EUR, EAS and AFR as reference. We set the number of upper clusters, lower clusters and mixture generations to 3, 15 and 200 as recommended by the software author. Inference was performed with 20 EM steps and averaged over ten replicates. The contribution of each of the three ancestral populations is given as an allele dosage estimate ranging from 0 to 2, corresponding respectively to no or complete contribution of the reference haplotype. Means for the region of interest were averaged across 1 kb up- and downstream from rs113332494. Mean dosage estimates of a single ancestral population and normed differences of two populations [POP1/(POP1 + POP2) − POP2/(POP1 + POP2)] were compared between groups. Permutation tests were used to derive the significance of group comparisons. We conducted 10,000 permutations shuffling group membership while keeping the group size constant.

Best guess genotypes with maximum posterior probability > 0.7 were used to determine the predictive power of rs113332494. The risks in the high- and low-risk group as well as positive and negative predictive value were calculated with respect to the assumed risk of agranulocytosis (0.9%) and neutropenia (3.8%)^9^. Individuals were classified as “high risk” when carrying the risk allele G, additionally local ancestry information was used in a second model.

Power calculations were performed with Quanto^37^ using a log-additive model. Power was derived using the estimated allele frequencies and case–control ratios setting the prevalence for neutropenia and agranulocytosis to 3.8 and 0.9%^9^.

SNP annotations, conservation scores and LD plots were derived with SNiPA^38^. For annotation of marker effects see https://snipa.helmholtz-muenchen.de/snipa3/index.php?task=supplement. Further details are provided in Supplementary Methods.

## Results

### Risk allele distribution

We examined rs113332494 risk allele (G) carriers and the distribution of risk alleles in individuals of different ancestries (*N* = 1574). The study sample was mainly comprised of individuals of European (*N* = 1004, 133 cases) ancestry, but also included individuals of African (*N* = 48, 15 cases) and admixed (*N* = 522, 30 cases) ancestry. Eight controls had maximum genotype posterior probabilities below the threshold (0.7) and were set to missing. We found 68 (4.3%) risk allele carriers in the 1566 remaining individuals, 52 (3.7% of 1396) controls and 16 (9.0% of 178) cases of which 9 (15.0% of 60) developed agranulocytosis. Fourteen cases were confirmed by neutrophil counts, whereas two cases (one neutropenia, one agranulocytosis) were confirmed by clinical judgement (blind to genotype). Of the 68 risk allele carriers, 66 had a diagnosis of schizophrenia, the other 2 had diagnoses of bipolar affective disorder and psychosis. We found 45 (15 cases) risk allele carriers of European, 6 (1 case) of African and 17 (0 cases) of mixed ancestry (Supplementary Fig. S3, Supplementary Table S3). The risk allele frequency for all 1566 individuals was 2.2%, which is in line with all non-Asian 1KG phase 1 populations (2.4%). Finnish (1.8%) and EUR (2.3%) frequencies also agree with the estimates of the reference populations (FIN = 1.6%, EUR = 2.5%). The risk allele in the European sample was three times more frequent in cases than in controls. In contrast, the risk allele was more common in African ancestry controls compared with cases. We found no admixed ancestry cases carrying the risk allele. All subsequent analyses were conducted in individuals of European ancestry only.

### Local ancestry of the *HLA-DQB1* region

**Fig. 1:**
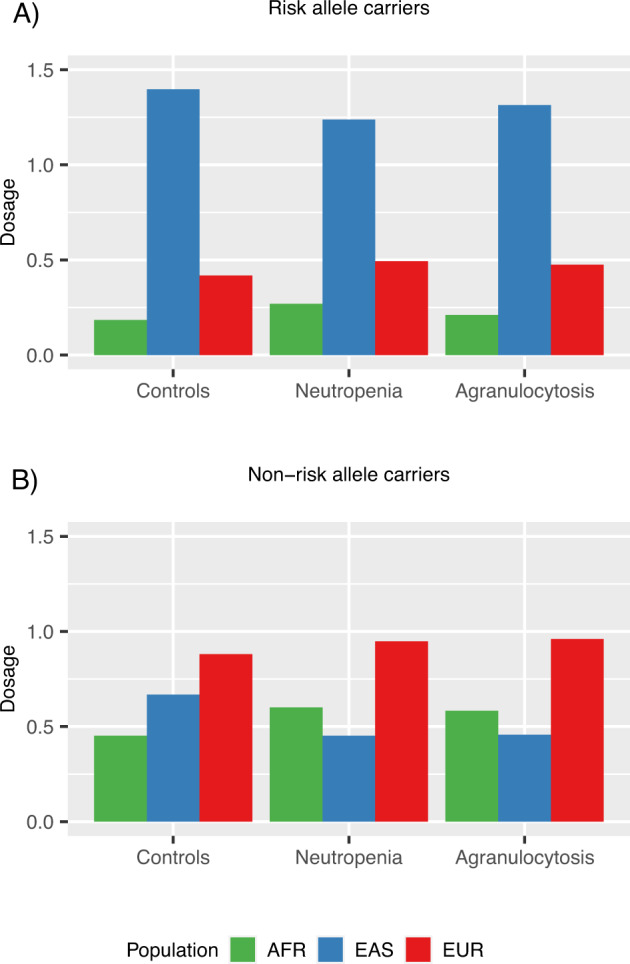
Local ancestry dosage estimates. Local ancestry dosage estimates of risk allele (**A**) and non-risk allele carriers (**B**) were estimated by ELAI using 1KG AFR, EAS and EUR super populations as reference (green = AFR, blue = EAS, red = EUR). Means for the region of interest were averaged across 1 kb up and downstream from rs113332494. Risk allele status was defined using best guess genotypes with posterior probability > 0.7.

We estimated local ancestry patterns in the extended MHC region and investigated the contribution of African, East Asian and European haplotypes in the region surrounding rs113332494 in individuals of European ancestry (*N* = 1004). In general, European dosage estimates dominated, but decreased in the *HLA-DQB1* region. Cases had higher African and lower East Asian local ancestry estimates than controls (Supplementary Fig. S4). Additional stratification by rs113332494 revealed two striking patterns. First, risk allele carriers exhibited considerably higher East Asian than European ancestry (EAS = 1.34, EUR = 0.44) (Fig. 1A, Supplementary Fig. S5A, C, E), whereas in non-carriers EUR local haplotypes dominated (EAS = 0.64, EUR = 0.89) (Fig. 1B, Supplementary Fig. S5B, D, F). Second, in the group of non-risk allele carriers, cases exhibited higher African and lower East Asian dosage estimates compared to controls (AFR controls = 0.45, AFR neutropenia = 0.60; EAS controls = 0.67, EAS neutropenia = 0.45) (Fig. 1B, Supplementary Fig. S5B, D, F). Patterns were similar for neutropenia and agranulocytosis cases.

The significance of group differences assessed by permutation testing is presented in Table 1. Risk allele carriers had significantly higher East Asian ancestry estimates than non-risk allele carriers (*P* < 1.00E−04). In addition, we found significant differences of East Asian (*P* < 1.00E−04) and African (*P* = 1.20E−03) ancestry in non-risk allele carriers between neutropenia cases and controls. The difference of AFR and EAS estimates also differed significantly between neutropenia cases and controls regardless of risk allele status, but this effect was driven by the difference in non-risk allele carriers, as there was no significant difference in risk allele carriers. Results were similar when restricting to agranulocytosis cases (Supplementary Tables S4 and S5).

**Table 1:** Local ancestry estimates in individuals of European ancestry for ancestral populations AFR, EAS and EUR.

Ancestral population	Non-risk vs risk allele carriers			Non-risk allele carriers: controls vs cases		
	Dosage	Difference	*P* value	dosage	difference	*P* value
AFR	0.470/0.213	0.257	**<1.00E−04**	0.452/0.600	−0.148	**1.20E−03**
EAS	0.641/1.344	−0.703	**<1.00E−04**	0.668/0.452	0.216	**<1.00E−04**
EUR	0.889/0.443	0.446	**<1.00E−04**	0.881/0.948	−0.067	8.31E−02
AFR–EAS	−0.154/−0.726	0.573	**<1.00E−04**	−0.193/0.141	−0.334	**<1.00E−04**
AFR–EUR	−0.308/−0.351	0.043	3.40E−01	−0.322/−0.224	−0.098	5.77E−02
EAS–EUR	−0.162/0.504	−0.666	**<1.00**E−**04**	−0.138/−0.353	0.216	**9.00**E−**04**

Dosage means for single ancestral populations and normed differences for two populations were compared between risk (*N* = 45) and non-risk allele (*N* = 956) carriers as well as between controls (*N* = 838) and neutropenia cases (*N* = 118) not carrying the risk allele. *P* values were determined by permutation tests (*N* = 10,000). Significant results are in bold.

### Association analyses

We performed an association analysis of the marker *HLA-DQB1* 6672G>C (rs113332494) in a European sample comprised of 871 controls, 133 neutropenia and 54 agranulocytosis cases. The risk allele was more frequent in cases (neutropenia = 5.4%, agranulocytosis = 8.0%, controls = 1.8%) and the variant was significantly associated with neutropenia (OR = 6.20, 95% CI: 2.91–13.21, *P* = 2.20E−06) and agranulocytosis (OR = 10.49, 95% CI: 3.99–27.56, *P* = 1.83E−06) (Table 2). Including AFR and EAS dosage estimates as additional covariates in association analyses gave higher odds ratio estimates (neutropenia: OR = 10.38, 95% CI: 4.45–24.22, *P* = 6.05E−08; agranulocytosis: OR = 16.31, 95% CI: 5.25–50.68, *P* = 1.39E−06), with the *P* value in the neutropenia analysis almost reaching genome-wide significance (Table 2). We had 100% power to detect effects in the size of the estimated odds ratios given the estimated allele frequency and case–control ratio. A sensitivity analysis including age and gender as covariates revealed associations and odds ratios of comparable strength and effect size (Supplementary Table S6).

**Table 2:** Results of association analyses of rs113332494 for neutropenia and agranulocytosis including 1004 and 925 individuals of European ancestry.

Phenotype	*N* cases	*N* controls	Info	Freq cases	Freq controls	Global ancestry		Global and local ancestry	
						OR 95% CI	*P* value	OR 95% CI	*P* value
Neutropenia	133	871	0.962	0.054	0.018	6.20	**2.20E−06**	10.38	**6.05E−08**
						2.91–13.21		4.45–24.22	
Agranulocytosis	54	871	0.969	0.080	0.018	10.49	**1.83E−06**	16.31	**1.39E−06**
						3.99–27.56		5.25–50.68	

Estimates are given for risk allele G. Global ancestry: association results corrected for principal components (PC1–PC7). Global and local ancestry: association results corrected for principal components (PC1–PC7) and local ancestry estimates for ancestral populations AFR and EAS. Significant results are in bold.

*Freq* frequency of risk allele G, *OR* odds ratio for risk allele G, *95%* *CI* 95% confidence interval for odds ratio.

### Linear effect of ANC

**Fig. 2:**
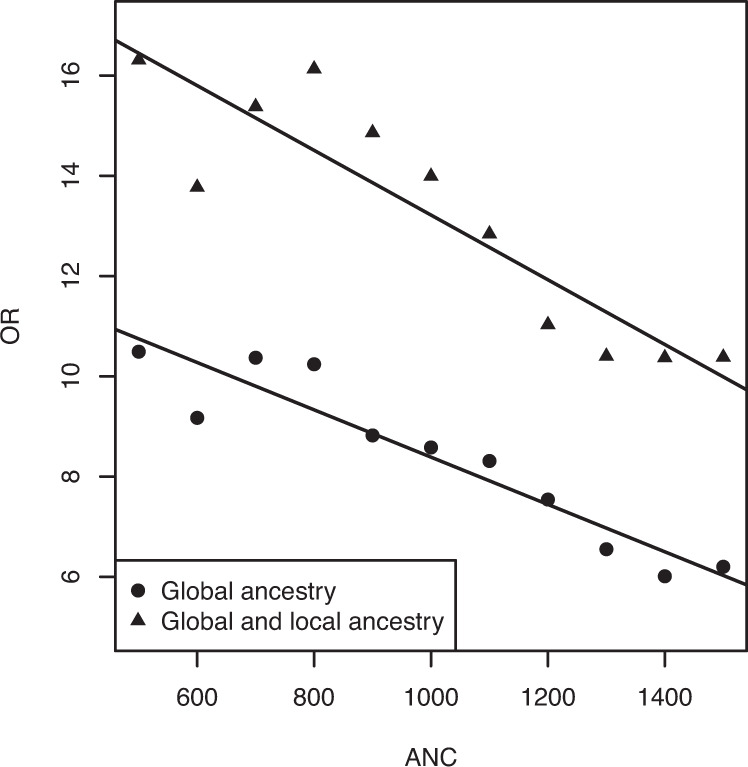
Odds ratio estimates for varying ANC thresholds (500–1500). Odds ratios were determined in association analyses of European individuals corrected for global (principal components PC1–PC7) and additionally for local ancestry (dosage estimates for ancestral populations AFR and EAS). Regression lines were determined by weighted least squares regression where the reciprocals of the standard errors were used as weights.

The odds ratio estimates were higher for agranulocytosis than for neutropenia. To determine if the effect on neutropenia is driven by inclusion of those with agranulocytosis, we excluded all cases with ANC ≥ 500 and tested a range of ANC thresholds (500–1500 in steps of 100) (Supplementary Table S7). The association diminished when excluding all agranulocytosis cases but was still significant, odds ratios decreased slightly with higher ANC thresholds (Supplementary Table S7, Supplementary Fig. S6). To determine how rs113332494 behaves as a predictor we also analysed different ANC thresholds excluding neutropenia cases for a range of ANC thresholds in steps of 100 (Supplementary Table S8). The odds ratios nearly followed a linear trend, where estimates decreased with higher ANC thresholds (Fig. 2).

### Predictive test characteristics

Predictive power was established by comparing affection to risk allele status, where heterozygote and homozygote risk allele carriers were combined. The *HLA-DQB1* variant had high specificity, but low sensitivity in predicting risk for agranulocytosis and neutropenia (Table 3). Sensitivity was higher in agranulocytosis (17%) cases in comparison to those with neutropenia (11%). Classifying all individuals carrying the rs113332494 risk allele as “high risk” and classifying non-risk allele carriers with respect to the difference in African and East Asian ancestry estimates (positive: higher AFR = “high risk”, negative: lower AFR = “low risk”) resulted in a substantial increase in sensitivity (neutropenia = 56%, agranulocytosis = 54%), but also a decrease in specificity (Table 3).

**Table 3:** Predictive test characteristics of rs113332494 including 868 controls and 133 neutropenia cases (ANC ≤ 1500) of which 54 developed agranulocytosis (ANC ≤ 500).

Phenotype	Neutropenia	Neutropenia	Agranulocytosis	Agranulocytosis
Risk Predictor	rs113332494	rs113332494 + LAE	rs113332494	rs113332494 + LAE
True positives	15	74	9	29
False positives	30	336	30	336
False negatives	118	59	45	25
True negatives	838	532	838	532
Sensitivity	0.1128	0.5564	0.1667	0.5370
Specificity	0.9654	0.6129	0.9654	0.6129
Risk in high-risk group	0.1142	0.0537	0.0420	0.0124
Risk in low-risk group	0.0350	0.0278	0.0078	0.0068
PPV	0.1142	0.0537	0.0420	0.0124
NPV	0.9650	0.9722	0.9922	0.9932
RR	0.3328	0.7073	0.2145	0.7233

Risk predictor = rs113332494: Individuals were classified as “high risk” when carrying the risk allele G. Genotype counts are based on best guess genotypes with maximum posterior probability > 0.7. Risk predictor = rs113332494 + local ancestry estimates: Individuals were classified as “high risk” when carrying the risk allele G. In addition, non-risk allele carriers were classified as “high risk” when they exhibited larger AFR dosage estimate compared to EAS. Risk of neutropenia and agranulocytosis were set to 0.009 and 0.038. The risks in the high- and low-risk group as well as positive and negative predictive value were calculated with respect to the assumed risk of agranulocytosis and neutropenia. The relative risk corresponds to the proportion of the assumed risk vs. the risk in the high-risk group.

*LAE* local ancestry estimate, *PPV* positive predictive value, *NPV* negative predictive value, *RR* relative risk.

## Discussion

Our study shows an association between rs113332494 (*HLA-DQB1* 6672G>C) and clozapine-induced neutropenia and agranulocytosis, further adding to the evidence of the importance of this genetic variant. The estimated odds ratio for agranulocytosis was higher (OR = 10.49, *P* = 1.83E−06) than for neutropenia (OR = 6.20, *P* = 2.20E−06). Exclusion of individuals with agranulocytosis revealed a residual, but diminished association signal, in those with neutropenia. Additional association analyses including cases with varying ANC thresholds (500–1500) showed that effect sizes increased linearly with stricter ANC thresholds. Most likely, this indicates that neutropenia cases are composed of two different populations: (1) individuals who would have developed agranulocytosis if clozapine had not been stopped and (2) individuals who show decreased ANC because of random fluctuation or other factors not related to clozapine-induced agranulocytosis. In this scenario, it might be the case that the number of “true” agranulocytosis cases increases as ANC counts decline.

**Fig. 3:**
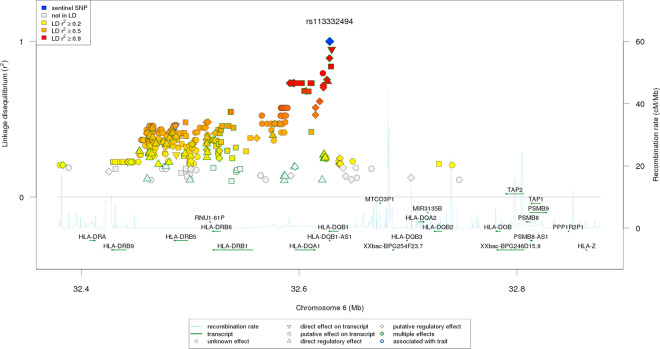
Linkage disequilibrium plot. Linkage disequilibrium of rs113332494 with surrounding markers coloured by strength of LD in 1 KG European super population.

Given the complex recombination pattern of the MHC region, it is unclear if rs113332494, which is intronic, is itself the causal variant rather than simply indicating an indirect association with another variant with which it is in LD. Annotations revealed three SNPs in strong LD (*r*^2^ > 0.8) to rs113332494 in the European population: rs41542812 (*r*^2^ = 0.95, direct transcript effect), rs113664950 (*r*^2^ = 0.89, putative regulatory/transcript effect) and rs114262759 (*r*^2^ = 0.84, putative transcript effect). The missense variant rs41542812 is a glutamine to histidine polymorphism at position 158. Rs113664950 is annotated as expressed promoter (FANTOM5) for *DQB1* and promoter-associated distal DHS (enhancer) for *PBX2, TAP1, HLA-DRB9, HLA-DQA1, HLA-DPA1, HLA-DQB1, HLA-DQA1, PBX2, HLA-DRB5, HLA-DOA* and *HLA-DPA1* (ENCODE). Interestingly, this variant is also associated with bare lymphocyte syndrome type I. Given the high LD between the investigated variant and rs113664950, an association of rs113332494 with this syndrome seems possible. One of twenty one variants in middle to high LD (0.6 < *r*^2^ < 0.8) showed a direct regulatory effect: rs35139945 (*r*^2^ > 0.74), which is a *cis*-eQTL variant for *HLA-DQA1*, *HLA-DRB5* and *HLA-DRB1*. Among other tissues differentially regulated, gene expression has been shown in blood^39^. LD decreases in the downstream direction of *HLA-DQB1* overlapping *HLA-DQA1* (*r*^2^ > 0.7) and the *HLA-DRB1*-*DRB6* (pseudo)-*DRB5* region (*r*^2^ > 0.4) in the European population (Fig. 3), whereas in African and Asian populations, the regions including high to low LD variants are more sharply limited surrounding the region of interest (Supplementary Fig. S7). Which variant(s) in this region is/are causal and whether variations in this region contribute to the risk in specific populations only remain subject to further investigation.

There is evidence that besides global population patterns, local haplotypes might be a relevant feature in the *HLA-DQB1* region. An excess of ancestry switches was reported for several populations and European ancestry was shown to diminish across the *HLA-DQB1* region in a British study on neutropenia. We determined the local haplotype structure in a sample without overlapping cases and also found decreasing European contribution to the region surrounding the marker rs113332494. The analysis revealed a substantial increase of East Asian local haplotypes in risk allele carriers, whereas in non-risk allele carriers European allele dosage estimates dominated. This pattern was present in cases and controls and thus did not provide additional information for risk detection or prediction. Interestingly, we also found significantly lower EAS and higher AFR dosage estimates in cases compared to controls not carrying the risk allele. Furthermore, including the local haplotype information substantially strengthened the association with both neutropenia (OR = 10.38, *P* = 6.05E−08) and agranulocytosis (OR = 16.31, *P* = 1.39E−06). However, local ancestry inferences should not be interpreted as conclusive assignments of haplotypes to specific populations^40^, as the resolution of the reference panel might not be enough to resolve the observed pattern of genetic diversity. In addition, it is not clear how the input sample or the choice of markers influences local ancestry estimates and if the effect is additive as was assumed in this study. Nevertheless, if further evaluated and replicated, these results could contribute to improve prediction of haematological side effects.

Individuals of African ancestry have a higher prevalence of BEN^14^. This study included individuals with >80% global European ancestry patterns. Thus, one might speculate that higher local African ancestry in those with low ANC might be related to global African ancestry patterns, and specifically, a higher propensity for BEN. However, local and global African ancestry do not correlate in non-risk allele carriers (controls: Kendall’s tau*-*b = 0.003, *P* = 0.89, neutropenia: Kendall’s tau*-*b = 0.024, *P* = 0.74), suggesting that the local effect is not driven by uncontrolled effects of global ancestry. Moreover, among all non-risk allele carriers, we found only one individual, who was actually a control, who was homozygous for the C-allele at SNP rs2814778, which is actually thought to be the causal genotype for BEN, and is a better predictor of BEN than global ancestry^15^. Thus, our finding appears to reflect local, not genome-wide, effects of African ancestry beyond those indexed by rs113332494. The observation of a local effect of African ancestry independent of rs113332494 suggests that there are either additional causal alleles within the region that are enriched in people of African ancestry, and/or that rs113332494 is not causal but is instead in LD with the causal variant.

To be of clinical use, a predictive pharmacogenetic test should be sufficient to identify individuals for which the risk of developing adverse effects is low enough to make blood monitoring either unnecessary or reduce the frequency by which it is performed. This corresponds to a low risk of developing adverse effects in the group of non-risk allele carriers. Verbelen et al. analysed the characteristics of a useful predictive variant and showed that the key factor is high sensitivity, especially in combination with a low frequency of risk allele carriers. Sensitivity or the true positive rate for a test denotes the proportion of individuals developing a side effect who are positive for the test, high sensitivity implying that the number of people falsely classified as being not at risk is low. In the context of high sensitivity, a low-risk allele frequency also implies that the absolute numbers of people correctly classified as being not at risk is larger, and thus blood monitoring can be relaxed for a large number of individuals. Assuming an acceptable risk of 0.13% (corresponding to the risk of chlorpromazine which does not require extensive blood monitoring in the UK) for agranulocytosis in the group of non-risk allele carriers and an agranulocytosis risk of 0.9%, sensitivity must be at least 85.7%^41^ in order to relax monitoring in those that are not positive for the test (i.e. do not have the high-risk allele). As in earlier studies, where sensitivity was found to be 21.5^21^ and 5%^25^, we failed to reach the required threshold. However, using local ancestry estimates for prediction, the sensitivity of rs113332494 increased from 11.28 to 55.64% for neutropenia and from 16.67 to 53.70% for agranulocytosis, thus coming closer to the goal of using variants as a predictive tool to give patients the chance of getting an effective treatment as soon as possible and with minimal disruption.

While our results show the importance of the marker under study, they should be viewed in light of some limitations. First of all, the sample size is arguably small, but this is unavoidable given the low prevalence of agranulocytosis. For this reason, the project allowed the inclusion of patients of all ancestries. However, the analysis of the risk allele distribution showed that we do not have enough samples to conduct an association analysis in individuals of African, Asian or mixed ancestry and thus is limited to individuals of European ancestry. Second, the current study is limited to one single SNP. The variant was not imputed with enough quality in other GWAs, but has been replicated in independent samples and is thus of special interest. Therefore, we conducted additional analyses for refinement of findings, while genome-wide analyses are underway. Third, different groups recruited patients under different conditions. While following standardized guidelines, some patients were recruited in hospitals during treatment, others were recruited from registers where the available information on clinical and demographic features was limited (see Supplementary Information). For example, treatment compliance was monitored and good compliance was defined as clinical impression > 80% of dosage and/or consistently documented plasma levels. However, plasma levels could not be included in the statistical analysis, as this information was not available for all study subjects. Last, the neutropenia sample might also include individuals who would have developed agranulocytosis if clozapine had not been stopped, but this follows the monitoring guidelines in countries of contributing groups and is the situation encountered in clinical practice.

In summary, we were able to replicate previous findings for an association of rs113332494 (*HLA-DQB1* 6672G>C) with clozapine-induced neutropenia and agranulocytosis in individuals of European ancestry. We showed that local haplotype structure might be an important factor when considering regions containing an excess of ancestry switches, as is the case for the MHC region, and that these patterns might be relevant for determining risk. These findings could lead to novel insights into the pathogenesis of neutropenia and agranulocytosis. Using local ancestry estimates for prediction increased the sensitivity of rs113332494. Further studies are required to clarify the role of the marker rs113332494 and to analyse the influence of global and local ancestry patterns.

## Supplementary information

Supplementary Information
