# Supplementary material for: HLA-DQB1 6672G>C (rs113332494) is associated with clozapine-induced neutropenia and agranulocytosis in individuals of European ancestry
Source: Transl Psychiatry. 2021 Apr 12;11:214. doi: 10.1038/s41398-021-01322-w (PMC8042025; doi:10.1038/s41398-021-01322-w)
Supplement: Supplementary file 1 — Supplementary Information [file 41398_2021_1322_MOESM1_ESM.docx]

# Supplementary Information

## Sample description CRESTAR

Individuals were recruited in the course of the CRESTAR project (https://cordis.europa.eu/project/rcn/101231_de.html). Any psychiatric diagnosis was allowed; the primary psychiatric diagnosis was schizophrenia. Any ancestry was allowed. Control samples were exposed to clozapine for at least one year and under this medication their absolute neutrophil count was never below 2000/mm^3^. Cases included in the neutropenia or agranulocytosis study must have had an ANC ≤ 500/mm^3^ or 1500/mm^3^ respectively or a defined haematological adverse drug reaction by clinical judgment. Differing criteria and additional comments and details on the recruitment can be found below. Sample characteristics are summarized in Supplementary Table S1 and S2. Informed consent was obtained from all participants. Studies were approved by the local ethics committee and carried out in accordance to the Declarations of Helsinki.

## Details of participating groups

### Breen, Gerome | London, England | IMPACT

The patients recruited to IMPACT were between 18 and 65 years of age, with the following ICD 10 psychiatric diagnoses: F20–F29 (schizophrenia, psychotic disorders and schizoaffective disorder), ICD 10 F31.2 (bipolar) and ICD 10 F32.3, F33.3 (depressive episode with psychotic symptoms). Exclusion criteria were as follows: learning disability, a physical health problem that would influence metabolic measures or substance use habits, pregnancy or less than 6 months postpartum or under intensive care [for further details, see ^1^]. A total of 186 patients provided consent for pre-randomisation blood for gene expression. In all, 152 remained following quality control and removal of outliers. Outliers were defined as those with insufficient clinical data, inadequate quality information or technical outliers. Out of 152 individuals, 104 had a diagnosis of schizophrenia, 19 had a diagnosis of bipolar disorder, 18 had a schizoaffective disorder and six had a depressive disorder with psychosis. The less common diagnoses included one individual with schizotypal disorder, one with delusional disorder and three with ‘other nonorganic psychosis’. Only 148 individuals had scores on the positive and negative symptom score. The mean positive and negative symptom score was 50±12. The distribution of drugs is not mutually exclusive as some individuals were receiving several antipsychotics or other medications.

Ethical approval was obtained from The Joint South London and Maudsley and The Institute of Psychiatry NHS Research Ethics Committee (REC ref no. 09/H080/41).

### Ingimarsson, Oddur | Reykjavik, Iceland | Landspitali-study of psychosis

The study is a part of a wider ongoing longitudinal study of psychotic disorders in the Landspitali University Hospital (LUH) department of psychiatry focusing on patients with schizophrenia and bipolar disorder. Patients have been recruited to the study in several waves from 1986–2014. The majority of inpatients and outpatients at LUH with schizophrenia have been invited to take part in the study. Almost all the patients in study have been admitted to LUH. Most of the patients were recruited in 2000–2004. All patients in the study had a confirmed diagnosis of schizophrenia or bipolar disorder according to the “Schedules for Affective Disorder and Schizophrenia-Lifetime version” (SADS-L).

To identify patients who had used clozapine, a keyword search in the electronic health record for the text strings “clozapin”, “closapin” and “Leponex” was performed. Leponex was the only brand name of clozapine in Iceland until the generic “Clozapine Actavis” was introduced to the market in May 2014. All patients’ medical notes where the clozapine keywords were identified were reviewed to confirm that clozapine had been used and the time frame when it was used.

We searched electronically all available results of blood measurements at LUH for neutrophil counts and the database in the regional hospital in Akureyri for keywords to find medical notes where neutropenia or agranulocytosis were mentioned. The complete medical notes, electronic as well as on paper, were reviewed in order to confirm the diagnosis.

The study was approved by the Icelandic National Bioethics Committee (FS-02-041(03-030)) and the Data Protection Authority (2009090737ÞS).

### Pirmohamed, Munir | Liverpool, England

CIAG (clozapine-induced agranulocytosis/granulocytopenia) cases developed agranulocytosis (absolute neutrophil count, ANC < 500 cells/mm^3^) or granulocytopenia (500 ≤ ANC ≤ 1,000 cells/mm^3^) while taking clozapine and without a known medical condition that increases risk for agranulocytosis (e.g., haematological disorder or autoimmune disease). We allowed any primary psychiatric diagnosis (although most subjects had schizophrenia or schizoaffective disorder) and any reported ancestry was allowed. Heterogeneity due to ANC threshold, diagnosis, and ancestry was addressed analytically. Subjects were identified in two ways. Opportunistic ascertainment was via a broad survey of colleagues to identify all worldwide samples in existing CIAG collections that allowed rapid and efficient identification of existing CIAG cases and controls exposed to clozapine without CIAG. This included cases from the International Severe Adverse Events Consortium (iSAEC, http://www.saeconsortium.org). Systematic assessment included identification of additional cases from the Novartis and Teva clozapine registries (https://www.clozarilcare.com/care, https://www.clozapineregistry.com), and the electronic pharmaceutical record from the Pennsylvania state hospital system. Briefly, we contacted the physician of record to confirm an episode of CIAG, the physician then obtained permission from the potential subject to release contact information, and we then contacted the subject to attempt recruitment into this study. We attempted to obtain and review medical records for all subjects. Using a standardized review form, we abstracted demographic data, clinical diagnosis, and clozapine dosage, exposure duration, and the lowest ANC. Two senior CIAC clinicians evaluated all available data to verify CIAG case status. Most DNA samples were extracted from peripheral venous blood; a subset of DNA samples were from lymphoblastoid cell lines or oral sources. Most samples were subjected to standard quality control procedures (DNA quantification, electrophoresis to assess degradation, and genotyping a “fingerprint” of 25 common SNPs using a Sequenom iPLEX panel). Genotype data confirmed that all subjects were independent and of European ancestry.

### Rietschel, Marcella | Bonn / Mannheim, Germany | MooDs

These German samples were collected by separate groups within the MooDS Consortium in Mannheim, Bonn and Munich. For the PGC analyses, the samples were combined by chip and ancestry. In Bonn/Mannheim, cases were ascertained as previously described ^2^. All participants gave written informed consent and the local ethics committees approved the human subjects’ protocols.

### Rujescu, Dan | Munich / Halle, Germany | PAGES

The PAGES sample is comprised of around 3000 controls, 1000 schizophrenia patients and 300 individuals with other psychiatric diagnoses. Individuals were recruited in Munich from 1997 to 2012 and ongoing recruitment is taking place in Halle.

Detailed medical and psychiatric histories including a clinical interview using the Structured Clinical Interview for Axis I and II DSM-IV Disorders (SCID) ^3,4^ were obtained for all patients. In addition, lifetime history of clozapine prescription was assessed as part of an extensive interview. Exclusion criteria included a history of head injury or neurological diseases. Clinical case notes were used to confirm neutropenia status, and lowest recorded neutrophil levels were collected. Cases were diagnosed to be suffering from neutropenia based on the observation of an absolute neutrophil count between 500 and 1,500 cells/mm^3^, and from agranulocytosis if a further decline with an ANC below 500 cells/mm^3^ was observed. Controls were selected for prescription of a minimal dose of 100mg clozapine per day for at least 1 year with no record of neutrophil counts below 2000 cells/ mm^3^.

### Tiihonen, Jari | Stockholm, Sweden

Schizophrenia or schizoaffective disorder patients diagnosed according to DSM-IV (Diagnostic and Statistical Manual of Mental Disorders) criteria were included in the study. Informed consent and approval by institutional ethics review board were obtained. Cases were diagnosed to be suffering from severe neutropenia (threatening agranulocytosis) based on the observation of a rapid decline in the ANC to < 1,500 cells mm^-3^ and from a definite agranulocytosis observing another decline (ANC ⩽ 500 cells mm^-3^) despite immediate discontinuation of clozapine. Controls were matched for age and sex and came from the same region of Finland as the cases. The distribution of age, gender and clozapine dose was not significantly different between cases and controls.

### van der Weide, Karen and van der Straaten, Tahar| Harderwijk and Leiden, The Netherlands

The study was conducted in the Dutch Psychiatric Hospital GGz Centraal location Meerkanten and in the Dutch Psychiatric Health Services Rivierduinen according to national guidelines. Measurement of the number of leukocytes was performed just before the start of clozapine treatment, once a week in the first 18 weeks and once a month until the end of treatment ^5^. Patient selection occurred based on the criteria for clozapine induced agranulocytosis (CIA) and neutropenia as described by the Food and Drug Administration ^5^. CIA patients were selected based on at least one neutrophil count ⩽500 μl^-1^. Neutropenia patients were selected on at least one neutrophil count between 500 μl^-1^ and 1500 μl^-1^ or two neutrophil counts < 2000 μl^-1^ during clozapine treatment. Patients were excluded when a neutrophil count < 2000 μl^-1^ was recorded before the start of clozapine treatment.

Control patients were selected for prescription of clozapine for at least 1 year, no record of either neutrophil counts < 2000 μl^-1^ or leukocyte counts < 4000 μl^-1^. In all groups patients aged under 18 were excluded, as were patients for whom DNA samples could not be obtained. For patients of Mental Health Services Rivierduinen, DNA samples are not routinely collected, therefore DNA was obtained after selection of the patients.

The Medical Research Ethics Committee in Amsterdam assessed the project and stated that it was not subject to further investigation under the Medical Research (Human Subjects) Act. The study has been approved by the research board (Innova) of Psychiatric Hospital GGz Centraal.

### Walters, James | Cardiff, Wales | CLOZUK1

CLOZUK1 comprises individuals who were prescribed clozapine in the United Kingdom and have a clinical diagnosis of treatment-resistant schizophrenia ^6,7^. The CLOZUK samples were acquired anonymously by the research team, in accordance with ethics permissions and the UK Human Tissue Act, in collaboration with Novartis, one of the UK suppliers of clozapine. Twelve months after sample acquisition, the research team was informed of those who had developed neutropenia while taking clozapine and, where available, the recorded lowest neutrophil counts of these individuals were supplied. Controls had received clozapine for a minimum of a year without developing an ANC ≤ 2000 cells/mm^3^.

### Walters, James | Cardiff, Wales | CLOZUK2

CLOZUK2 comprises of individuals prescribed clozapine in the UK with a clinicial diagnosis of treatment-resistant schizophrenia. The samples were acquired in partnership with Leyden Delta (Nijmegen, Netherlands), a major UK supplier of clozapine, as part of the CRESTAR collaborative project (www.crestar-project.eu). The project has received UK National Research Ethics Service approval and was in accordance with the UK Human Tissue Act. Full details of the CLOZUK2 sample are provided elsewhere ^8^. All samples were anonymised and linked with blood monitoring data provided from clozapine blood monitoring databases. The lowest absolute neutrophil count (ANC) during clozapine treatment on record within the blood-monitoring database was provided. Any ANC < 1500 cells/mm^3^ were confirmed with either a consecutive ‘red’ ANC < 1500 cells/mm^3^ or a series of ‘amber’ ANC < 2000 cells/mm^3^. In addition, individuals with a clinician reported alternative possible reason for an abnormal ANC such as concomitant immunosuppressive medication were excluded.

### Walters, James | Cardiff, Wales | CardiffCOGs (Cognition in Schizophrenia)

CardiffCOGS is a schizophrenia sample recruited from secondary mental health services in South Wales, UK; for detailed sample description see ^6,9^. As part of a comprehensive clinical interview, individuals were asked about lifetime clozapine use and occurrence of neutropenia. Clinical case notes were used to confirm neutropenia status, and lowest recorded neutrophil levels were collected. Controls had received clozapine for a minimum of a year without developing an ANC ≤ 2000 cells/mm^3^.

## Genotyping, quality control and imputation

Individuals were genotyped on Illumina HumanOmniExpress-12v1-1_B at deCODE genetics. PLINK 1.9 ^10,11^ was used to screen for gender mismatches, autosomal heterozygosity and related individuals applying standard quality control (SNP missingness < 0.05, sample callrate ≥ 0.98, SNP missingness < 0.01, Hardy-Weinberg equilibrium (HWE) P > 1e-05, minor allele frequency (MAF) ≥ 0.01). For cryptic relatedness and heterozygosity analyses markers (MAF ≥ 0.01, HWE P < 0.05) were pruned after excluding several high LD regions as the major histocompatibility complex (MHC) on chromosome 6, retaining every marker in a window of 1500 variants removing one of a pair in LD greater than 0.2 shifting the window by 150 markers. All individuals failing sex-check or showing autosomal heterozygosity deviation (|Fhet| > 0.2) were excluded. Heterozygosity was analysed in several clusters to account for population structure. To get an unrelated set of individuals one of each pair having 𝜋̂ > 0.2 was excluded, where cases were retained over controls. 1576 individuals passed the quality control procedure.

Since minor allele frequencies vary across populations the final set of markers for pre-shaping and imputation was identified by applying a less stringent minor allele frequency threshold (MAF ≤ 0.0007, with at least 1 individual having the minor allele) and no HWE filter on the pre-defined set of reliable unrelated individuals. It has been shown that imputation quality depends more on the number of markers and percentage of missingness than on filter criterions as minor allele frequency and HWE and that pre-imputation quality-filtering of SNPs results in highly similar imputation quality compared with no filtering ^12,13^. All other thresholds were set as described above.

SHAPEIT2 ^14^ and IMPUTE2 ^15,16^ were used for pre-shaping and imputation using the full 1000 Genomes (1KG) reference panel phase 1 version 3 macGT1 (August 2012). Individuals of all ancestries were shaped at once ^17^. Pre-shaping was applied on each chromosome arm setting the effective population size to 17,469. Imputation was performed in chunk sizes of mainly 5MB where chunks were extended up to ~7MB if otherwise small sized intervals (<5MB) would have been left.

Sample imputation quality differed between populations where African individuals had lowest concordance rates with masked genotyped markers of ~93 - 95%. The Finnish individuals exhibited the highest concordance rates (>98%). Rates of 95 - 98% could be achieved for all European individuals and those of admixed ancestries. This is in line with reported concordance rates of other studies. Mean concordance over all chunks and markers was 97%.

## Population structure

Common markers (MAF > 0.05) with high genotyping success (missingness < 0.99) and in HWE (P > 0.05) were pruned by PLINK after excluding high LD regions as the major histocompatibility complex (MHC) on chromosome 6 retaining every marker in a window of 1500 variants removing one of a pair in LD greater than 0.2 shifting the window by 150 markers. The pruned set of markers was used for principal components and admixture analyses conducted with EIGENSTRAT v6.0.1 ^18^ and ADMIXTURE v1.3.0 ^19^.

Genotypes were merged with 1000 Genomes Phase 1 data ^20^ (available at https://www.cog-genomics.org/plink2/resources#1kg) and filtered as described above. We defined European and African subsamples on estimated ancestry fractions (>80%) determined by a supervised ADMIXTURE analysis using 1KG populations AFR, EAS and EUR as reference. The CRESTAR sample was mainly comprised of individuals of European ancestry, but also included individuals of African and admixed ancestry (Supplementary Figure S1). Self-described ancestry matched where available.

EIGENSTRAT analysis was used to remove outliers and calculate principal components for individuals of European ancestry. Eigenvectors were screened for outliers defined as six standard deviations from the mean using five iterations (evec = 10, iter = 5, sigma = 6). EIGENSTRAT outlier removal was performed separately on Finnish and non-Finnish individuals, as the Finnish and other European populations formed two distinct clusters (Supplementary Figure S2). Estimated ancestry fractions for Finnish individuals exhibited a larger East Asian and a lower European and African background (AFR = 0%, EAS = 7%, EUR = 93%) compared to the non-Finnish Europeans (AFR = 2%, EAS = 1%, EUR = 97%). The European ancestry fraction of both groups was 97% on average. Principal components for statistical analyses were derived on the combined sample.

## Statistical analyses

Association analyses in individuals of European ancestry were performed using logistic regression in PLINK ^10,11^ applying an additive model. Principal components were derived on controls and neutropenia cases and were also used in the analysis of stricter ANC thresholds. Scree plots of eigenvalues were screened for relevant principal components and the first 10 principal components were tested for association with phenotype. We identified six relevant components. PC7 was associated with neutropenia and agranulocytosis and additionally included in the logistic model. Scatterplots of principal components 1 and 2 are given in Supplementary Figure S1 and S2.

## Overlap to other studies

Overlap between cases of CRESTAR and CLOZUK ^21^ was determined by examining ~1000 markers randomly sampled from well-imputed markers used for polygenic risk score prediction in the PGC schizophrenia meta-analysis (<https://www.med.unc.edu/pgc/results-and-downloads>) for identity-by-descent estimations. Simulations and known relationships showed that the number of markers is sufficient to identify duplicates and first-grade relationships, thus we excluded all cases with 𝜋̂ > 0.4. One neutropenia and one agranulocytosis case were excluded for all statistical analyses since they were found to be duplicates in the CLOZUK study.

Overlap to CIAC ^22^ was determined by identity-by-descent estimations examining a pruned set of genome-wide markers in controls and cases. We identified 15 individuals (8 controls, 7 cases) of European ancestry as duplicates. As the studied variant was not imputed with enough quality in CIAC, we decided to keep the overlapping individuals in our statistical analyses. For completeness, we ran association analyses excluding the 15 individuals and the association signals and effect sizes were of similar magnitude:

- Neutropenia (863 controls, 126 cases)
  - Global ancestry: OR = 5.82, 95% CI: 2.64-12.85, P = 1.27E-05
  - Global and local ancestry: OR = 9.75, 95% CI: 4.02-23.62, P = 4.64E-07
- Agranulocytosis (863 controls, 51 cases)
  - Global ancestry: OR = 10.35, 95% CI: 3.75-28.57, P = 6.39E-06
  - Global and local ancestry: OR = 16.31, 95% CI: 4.93-54.00, P = 4.87E-06

## Supplementary Tables

#### Supplementary Table S1: Sample size by study group.

| **Group** | **Controls**  **N** | **Neutropenia N (report)** | **Agranulocytosis**  **N** |
| --- | --- | --- | --- |
| Bonn/Mannheim | 0 | 4 (3) | 0 |
| CardiffCOGs | 65 | 5 (4) | 0 |
| CLOZUK1 | 46 | 7 (1) | 0 |
| CLOZUK2 | 945 | 23 | 2 |
| Finland | 30 | 24 | 13 |
| Halle/Munich | 165 | 13 (1) | 7 |
| Liverpool | 114 | 49 | 13 |
| London | 0 | 6 (3) | 0 |
| Netherlands | 31 | 49 (7) | 26 |
| All | 1396 | 180 (19) | 61 |

#### Supplementary Table S2: Age and gender characteristics of the CRESTAR sample.

|  | **N** | **Male gender**  **N (%)** | **Mean Age**  **(range)^1^** |
| --- | --- | --- | --- |
| Controls | 1396 | 998 (71.5) | 35.4 (14-72) |
| Neutropenia | 180 | 132 (73.3) | 36.3 (16-79) |
| Agranulocytosis | 61 | 41 (67.2) | 41.0 (16-79) |
| All | 1576 | 1130 (71.7) | 35.5 (14-79) |

^1^If available age is defined as the treatment start; otherwise, age is estimated by other available information. Age was missing for one individual.

#### Supplementary Table S3: Numbers of risk allele carriers in the CRESTAR sample. Risk allele carriers were defined on best guess genotypes with posterior probability > 0.7. N: Number of individuals in a group with given ancestry. Missings: Genotype set to missing. Homozygous: Number of individuals homozygous for the risk allele.

| **Group** | **Ancestry** | **N** | **Missings** | **Risk-allele carriers** | **Homozygous** |
| --- | --- | --- | --- | --- | --- |
| All individuals | AFR | 48 | 0 | 6 | 0 |
|  | EUR | 1004 | 3 | 45 | 1 |
|  | Other | 522 | 5 | 17 | 1 |
|  | All | 1574 | 8 | 68 | 2 |
| Controls | AFR | 33 | 0 | 5 | 0 |
|  | EUR | 871 | 3 | 30 | 1 |
|  | Other | 492 | 5 | 17 | 1 |
|  | All | 1396 | 8 | 52 | 2 |
| Neutropenia | AFR | 15 | 0 | 1 | 0 |
|  | EUR | 133 | 0 | 15 | 0 |
|  | Other | 30 | 0 | 0 | 0 |
|  | All | 178 | 0 | 16 | 0 |
| Agranulocytosis | AFR | 1 | 0 | 0 | 0 |
|  | EUR | 54 | 0 | 9 | 0 |
|  | Other | 5 | 0 | 0 | 0 |
|  | All | 60 | 0 | 9 | 0 |

#### Supplementary Table S4: Local ancestry estimates in individuals of European ancestry for ancestral populations AFR, EAS and EUR. Dosage means of single ancestral populations were compared between groups stratified by risk allele and case-control status. P-values were determined by permutation tests (N = 10 000). Significant results are in bold.

| **Comparison** | **Phenotype** | **N** |  | **AFR** | |  | **EAS** | |  | **EUR** | |
| --- | --- | --- | --- | --- | --- | --- | --- | --- | --- | --- | --- |
|  |  |  |  | **Dosage** | **P-value** |  | **Dosage** | **P-value** |  | **Dosage** | **P-value** |
| Controls vs. cases | Neutropenia | 871 / 133 |  | 0.442 / 0.563 | **5.60E-03** |  | 0.695 / 0.541 | **7.00E-04** |  | 0.863 / 0.896 | 2.31E-01 |
|  | Agranulocytosis | 871 / 54 |  | 0.442 / 0.521 | 1.28E-01 |  | 0.695 / 0.600 | 1.01E-01 |  | 0.863 / 0.880 | 4.00E-01 |
| Non-risk vs. risk-allele carriers | All | 956 /45 |  | 0.470 / 0.213 | **<1.00E-04** |  | 0.641 / 1.344 | **<1.00E-04** |  | 0.889 / 0.443 | **<1.00E-04** |
|  | Controls | 838 / 30 |  | 0.452 / 0.185 | **5.00E-04** |  | 0.668 / 1.379 | **<1.00E-04** |  | 0.881 / 0.418 | **<1.00E-04** |
|  | Neutropenia | 118 / 15 |  | 0.600 / 0.269 | **9.50E-03** |  | 0.452 / 1.238 | **<1.00E-04** |  | 0.948 / 0.493 | **6.00E-04** |
|  | Agranulocytosis | 45 / 9 |  | 0.583 / 0.211 | **3.90E-02** |  | 0.457 / 1.314 | **<1.00E-04** |  | 0.960 / 0.475 | **8.10E-03** |
| Controls vs. cases in non-risk allele carriers | Neutropenia | 838 / 118 |  | 0.452 / 0.600 | **1.20E-03** |  | 0.668 / 0.452 | **<1.00E-04** |  | 0.881 / 0.948 | 8.31E-02 |
|  | Agranulocytosis | 838 / 45 |  | 0.452 / 0.583 | **4.38E-02** |  | 0.668 / 0.457 | **2.80E-03** |  | 0.881 / 0.960 | 1.40E-01 |
| Controls vs. cases in risk allele carriers | Neutropenia | 30 / 15 |  | 0.185 / 0.269 | 2.05E-01 |  | 1.397 / 1.238 | 1.00E-01 |  | 0.418 / 0.493 | 2.57E-01 |
|  | Agranulocytosis | 30 / 9 |  | 0.185 / 0.269 | 2.05E-01 |  | 1.397 / 1.238 | 1.00E-01 |  | 0.418 / 0.493 | 2.57E-01 |

#### Supplementary Table S5: Local ancestry estimates in individuals of European ancestry for ancestral populations AFR, EAS and EUR. Normed differences of dosage means were compared between groups stratified by risk allele and case-control status. P-values were determined by permutation tests (N = 10 000). Significant results are in bold.

| **Comparison** | **Phenotype** | **N** |  | **AFR-EAS** | |  | **AFR-EUR** | |  | **EAS-EUR** | |
| --- | --- | --- | --- | --- | --- | --- | --- | --- | --- | --- | --- |
|  |  |  |  | **Dosage** | **P-value** |  | **Dosage** | **P-value** |  | **Dosage** | **P-value** |
| Controls vs. cases | Neutropenia | 871 / 133 |  | -0.222 / 0.020 | **5.00E-04** |  | -0.322 / -0.228 | 5.51E-02 |  | -0.108 / -0.248 | **4.70E-03** |
|  | Agranulocytosis | 871 / 54 |  | -0.222 / -0.070 | 8.80E-02 |  | -0.322 / -0.256 | 2.30E-01 |  | -0.108 / -0.189 | 1.61E-01 |
| Non-risk vs. risk-allele carriers | All | 956 /45 |  | -0.154 / -0.726 | **<1.00E-04** |  | -0.308 / -0.351 | 3.40E-01 |  | -0.162 / 0.504 | **<1.00E-04** |
|  | Controls | 838 / 30 |  | -0.193 / -0.766 | **<1.00E-04** |  | -0.322 / -0.387 | 2.93E-01 |  | -0.138 / 0.539 | **<1.00E-04** |
|  | Neutropenia | 118 / 15 |  | 0.141 / -0.643 | **<1.00E-04** |  | -0.224 /- 0.293 | 3.56E-01 |  | -0.354 / 0.430 | **<1.00E-04** |
|  | Agranulocytosis | 45 / 9 |  | 0.121 / -0.723 | **3.10E-03** |  | -0.245 / -0.385 | 3.32E-01 |  | -0.356 /0.469 | **<1.00E-04** |
| Controls vs. cases in non-risk allele carriers | Neutropenia | 838 / 118 |  | -0.193 / 0.141 | **<1.00E-04** |  | -0.322 / -0.224 | 5.77E-02 |  | -0.138 / -0.354 | **3.00E-04** |
|  | Agranulocytosis | 838 / 45 |  | -0.193 / 0.121 | **5.20E-03** |  | -0.322 / -0.245 | 2.08E-01 |  | -0.138 / -0.356 | **5.50E-03** |
| Controls vs. cases in risk allele carriers | Neutropenia | 30 / 15 |  | -0.766 / -0.643 | 1.64E-01 |  | -0.387 / -0.293 | 3.66E-01 |  | 0.539 / 0.430 | 1.84E-01 |
|  | Agranulocytosis | 30 / 9 |  | -0.766 / -0.723 | 3.83E-01 |  | -0.387 / -0.385 | 4.93E-01 |  | 0.539 / 0.469 | 3.09E-01 |

#### Supplementary Table S6: Comparison of logistic association results of rs113332494 for neutropenia and agranulocytosis in individuals of European ancestry corrected for global (PC1-PC7) and local ancestry (estimates for ancestral populations AFR and EAS) with results additionally corrected for age and gender. Significant results are in bold.

| **Phenotype** | **Ancestry correction** |  | **Without age and gender** | | |  | **With age and gender** | | |
| --- | --- | --- | --- | --- | --- | --- | --- | --- | --- |
|  |  |  | **OR** | **SE** | **P-value** |  | **OR** | **SE** | **P-value** |
| Neutropenia | Global ancestry |  | 6.20 | 0.39 | **2.20E-06** |  | 6.27 | 0.39 | **2.36E-06** |
| Neutropenia | Global and local ancestry |  | 10.38 | 0.43 | **6.05E-08** |  | 10.40 | 0.43 | **7.17E-08** |
| Agranulocytosis | Global ancestry |  | 10.49 | 0.49 | **1.83E-06** |  | 10.81 | 0.51 | **2.87E-06** |
| Agranulocytosis | Global and local ancestry |  | 16.31 | 0.58 | **1.39E-06** |  | 15.80 | 0.59 | **2.46E-06** |

*OR* Odds ratio for risk allele G, *SE* Standard error

#### Supplementary Table S7: Results of association analyses of rs113332494 for varying ANC thresholds including individuals of European ancestry only (N controls = 871, Freq. controls = 0.018), excluding all agranulocytosis cases. Column ANC gives the lowest absolute neutrophil count not included; e.g. ANC = 500 includes all cases with ANC = 501-1500. Estimates are given for risk allele G. Estimates are not given for ANC thresholds 1300 and 1400 as models could not be fitted due to low sample size and resulting multicollinearity issues. Global ancestry: Association results corrected for principal components (PC1 - PC7). Global and local ancestry: Association results corrected for principal components (PC1 - PC7) and local ancestry estimates for ancestral populations AFR and EAS.

| **ANC** | **N cases** | **Info** | **Freq cases** |  | **Global ancestry** | |  | **Global and local ancestry** | |
| --- | --- | --- | --- | --- | --- | --- | --- | --- | --- |
|  |  |  |  |  | **OR CI 95%** | **P-value** |  | **OR CI 95%** | **P-value** |
| 500 | 79 | 0.970 | 0.0361 |  | 4.01 | 6.220E-03 |  | 7.00 | 5.044E-04 |
|  |  |  |  |  | 1.48 - 10.86 |  |  | 2.34 - 20.97 |  |
| 600 | 69 | 0.970 | 0.0413 |  | 4.54 | 2.876E-03 |  | 7.56 | 3.245E-04 |
|  |  |  |  |  | 1.68 - 12.28 |  |  | 2.51 - 22.79 |  |
| 700 | 66 | 0.972 | 0.0363 |  | 3.75 | 1.413E-02 |  | 6.35 | 1.805E-03 |
|  |  |  |  |  | 1.31 - 10.80 |  |  | 1.99 - 20.27 |  |
| 800 | 64 | 0.972 | 0.0374 |  | 3.77 | 1.379E-02 |  | 6.03 | 2.353E-03 |
|  |  |  |  |  | 1.31 - 10.84 |  |  | 1.89 - 19.20 |  |
| 900 | 59 | 0.972 | 0.0406 |  | 4.11 | 9.169E-03 |  | 6.36 | 1.960E-03 |
|  |  |  |  |  | 1.42 - 11.91 |  |  | 1.97 - 20.52 |  |
| 1000 | 54 | 0.973 | 0.0355 |  | 3.70 | 2.711E-02 |  | 5.69 | 7.446E-03 |
|  |  |  |  |  | 1.16 - 11.81 |  |  | 1.59 - 20.36 |  |
| 1100 | 47 | 0.975 | 0.0308 |  | 3.21 | 8.062E-02 |  | 5.43 | 2.097E-02 |
|  |  |  |  |  | 0.87 - 11.86 |  |  | 1.29 - 22.81 |  |
| 1200 | 37 | 0.975 | 0.0391 |  | 3.67 | 5.357E-02 |  | 7.40 | 9.128E-03 |
|  |  |  |  |  | 0.98 - 13.72 |  |  | 1.64 - 33.34 |  |

*Freq* Frequency of risk allele G, *OR* Odds ratio for risk allele G, *CI 95%* 95% confidence interval for odds ratio

#### Supplementary Table S8: Results of association analyses of rs113332494 for varying ANC thresholds including individuals of European ancestry (N controls = 871, Freq. controls = 0.018). Estimates are given for risk allele G. Column ANC gives the largest absolute neutrophil count included. Global ancestry: Association results corrected for principal components (PC1 - PC7). Global and local ancestry: Association results corrected for principal components (PC1 - PC7) and local ancestry estimates for ancestral populations AFR and EAS.

| **ANC** | **N cases** | **Info** | **Freq cases** |  | **Global ancestry** | |  | **Global and local ancestry** | |
| --- | --- | --- | --- | --- | --- | --- | --- | --- | --- |
|  |  |  |  |  | **OR CI 95%** | **P-value** |  | **OR CI 95%** | **P-value** |
| 500 | 54 | 0.967 | 0.080 |  | 10.49 | 1.832E-06 |  | 16.31 | 1.387E-06 |
|  |  |  |  |  | 3.99 - 27.56 |  |  | 5.25 - 50.68 |  |
| 600 | 57 | 0.967 | 0.076 |  | 9.17 | 4.451E-06 |  | 13.77 | 3.137E-06 |
|  |  |  |  |  | 3.56 - 23.64 |  |  | 4.57 - 41.48 |  |
| 700 | 60 | 0.965 | 0.080 |  | 10.37 | 7.445E-07 |  | 15.38 | 6.873E-07 |
|  |  |  |  |  | 4.11 - 26.19 |  |  | 5.23 - 45.27 |  |
| 800 | 62 | 0.965 | 0.077 |  | 10.24 | 9.163E-07 |  | 16.13 | 5.064E-07 |
|  |  |  |  |  | 4.04 - 25.91 |  |  | 5.45 - 47.74 |  |
| 900 | 67 | 0.965 | 0.071 |  | 8.82 | 2.692E-06 |  | 14.86 | 6.257E-07 |
|  |  |  |  |  | 3.55 - 21.89 |  |  | 5.14 - 42.94 |  |
| 1000 | 72 | 0.964 | 0.073 |  | 8.58 | 1.343E-06 |  | 13.99 | 3.084E-07 |
|  |  |  |  |  | 3.59 - 20.50 |  |  | 5.09 - 38.42 |  |
| 1100 | 79 | 0.963 | 0.073 |  | 8.31 | 6.970E-07 |  | 12.84 | 1.910E-07 |
|  |  |  |  |  | 3.60 - 19.16 |  |  | 4.91 - 33.54 |  |
| 1200 | 89 | 0.963 | 0.064 |  | 7.54 | 1.780E-06 |  | 11.03 | 5.070E-07 |
|  |  |  |  |  | 3.29 - 17.26 |  |  | 4.32 - 28.12 |  |
| 1300 | 102 | 0.962 | 0.061 |  | 6.55 | 2.990E-06 |  | 10.40 | 2.650E-07 |
|  |  |  |  |  | 2.98 - 14.42 |  |  | 4.26 - 25.35 |  |
| 1400 | 113 | 0.963 | 0.055 |  | 6.01 | 7.040E-06 |  | 10.37 | 2.190E-07 |
|  |  |  |  |  | 2.75 - 13.15 |  |  | 4.28 - 25.13 |  |
| 1500 | 133 | 0.961 | 0.054 |  | 6.20 | 2.204E-06 |  | 10.38 | 6.051E-08 |
|  |  |  |  |  | 2.91 - 13.21 |  |  | 4.45 - 24.22 |  |

*Freq* Frequency of risk allele G, *OR* Odds ratio for risk allele G, *CI 95%* 95% confidence interval for odds ratio

## Supplementary Figures


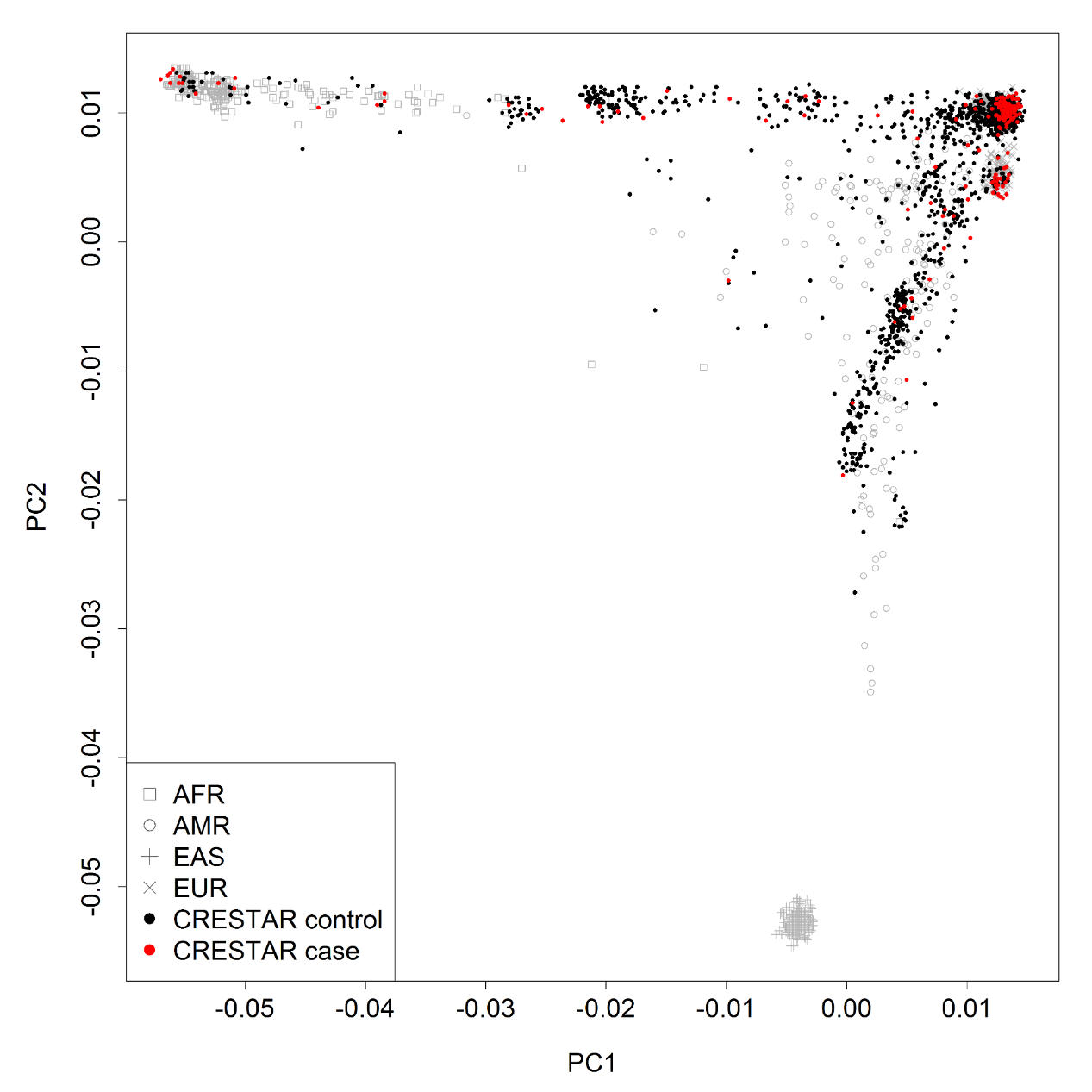


#### Supplementary Figure S1: Principal components 1 and 2 of EIGENSTRAT analysis based on CRESTAR individuals merged with 1000 Genomes data. Black points represent clozapine-treated controls and red points represent neutropenia cases. AFR populations are located top left, EUR populations top right, EAS populations bottom right and AMR populations are spread around and below the EUR populations.


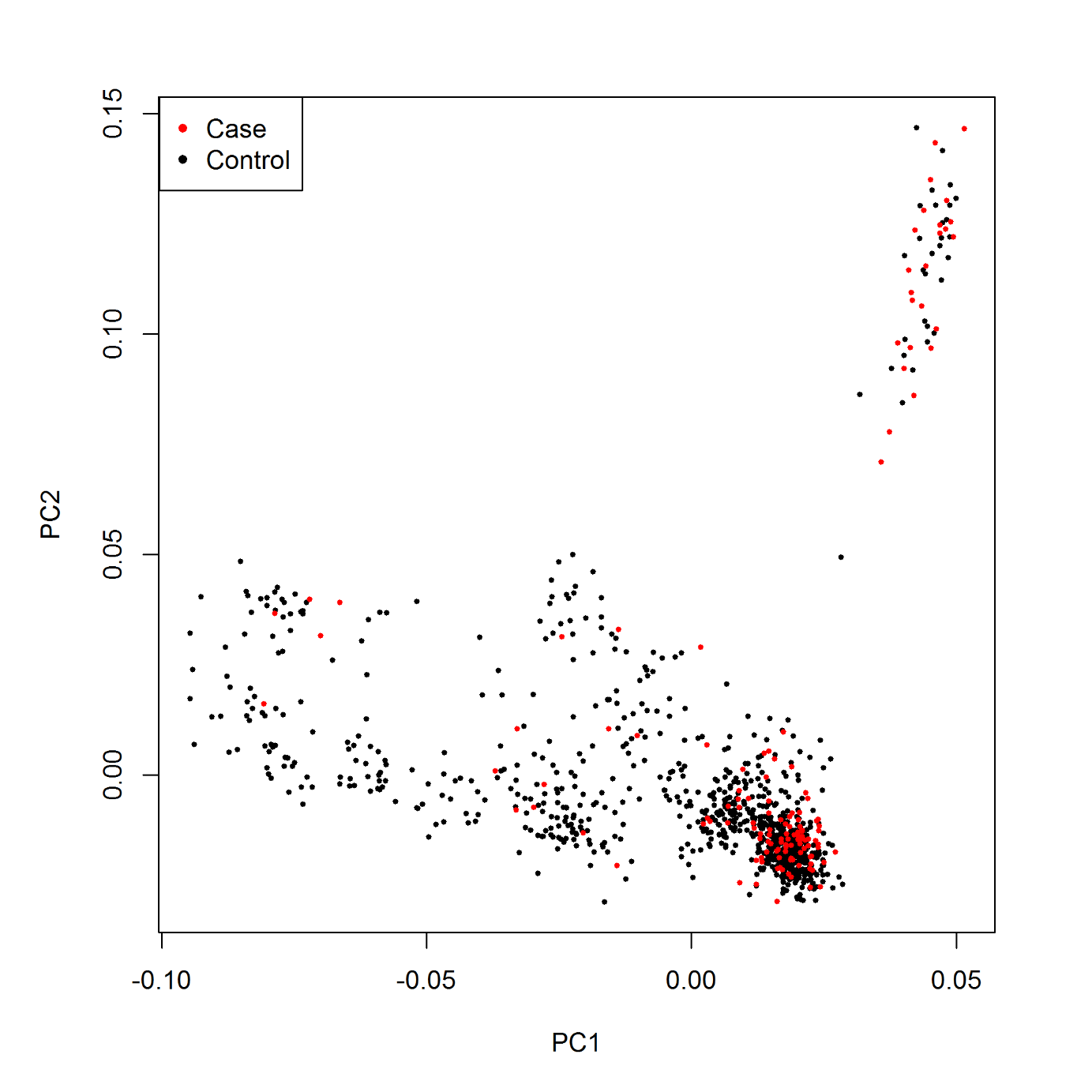


#### Supplementary Figure S2: Principal component 1 and 2 derived by EIGENSTRAT analysis on individuals with estimated European ancestry fractions > 80%. The Finnish population clustered separately (top right). Cases are well matched by controls.


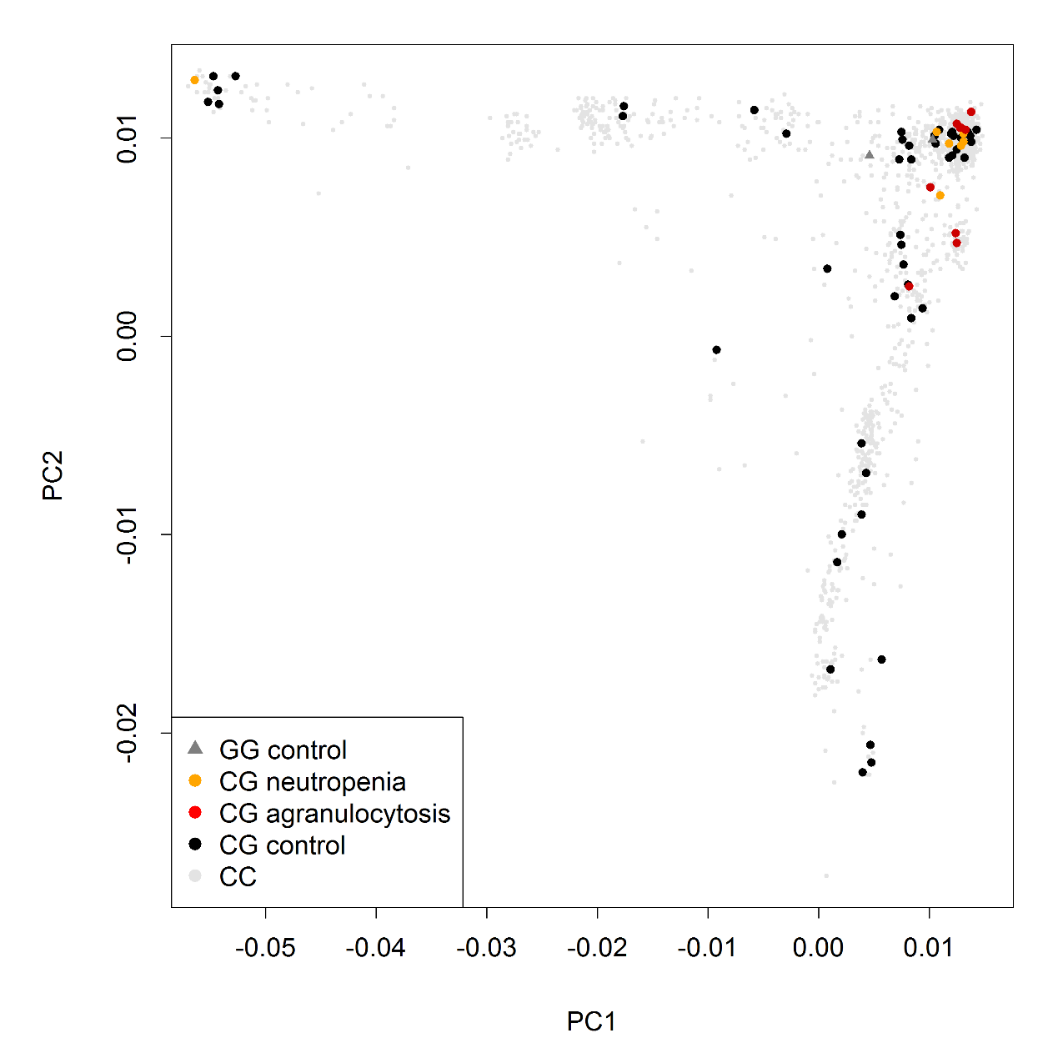


#### Supplementary Figure S3: Distribution of risk alleles in the CRESTAR sample. The scatterplot shows the first two principal components colored by genotype and status. Heterozygote and homozygote controls are colored black and dark-grey. Neutropenia and agranulocytosis cases are colored orange and red; please note that neutropenia does not include agranulocytosis. Triangles represent homozygous risk allele carriers and yellow, red, and black points represent heterozygous risk allele carriers. Small gray dots represent all other individuals. Risk alleles were determined on best guess genotypes with maximum posterior probability > 0.7. Individuals of African and European ancestry are located top left and right. Individuals show increasing East Asian ancestry from top to bottom right.


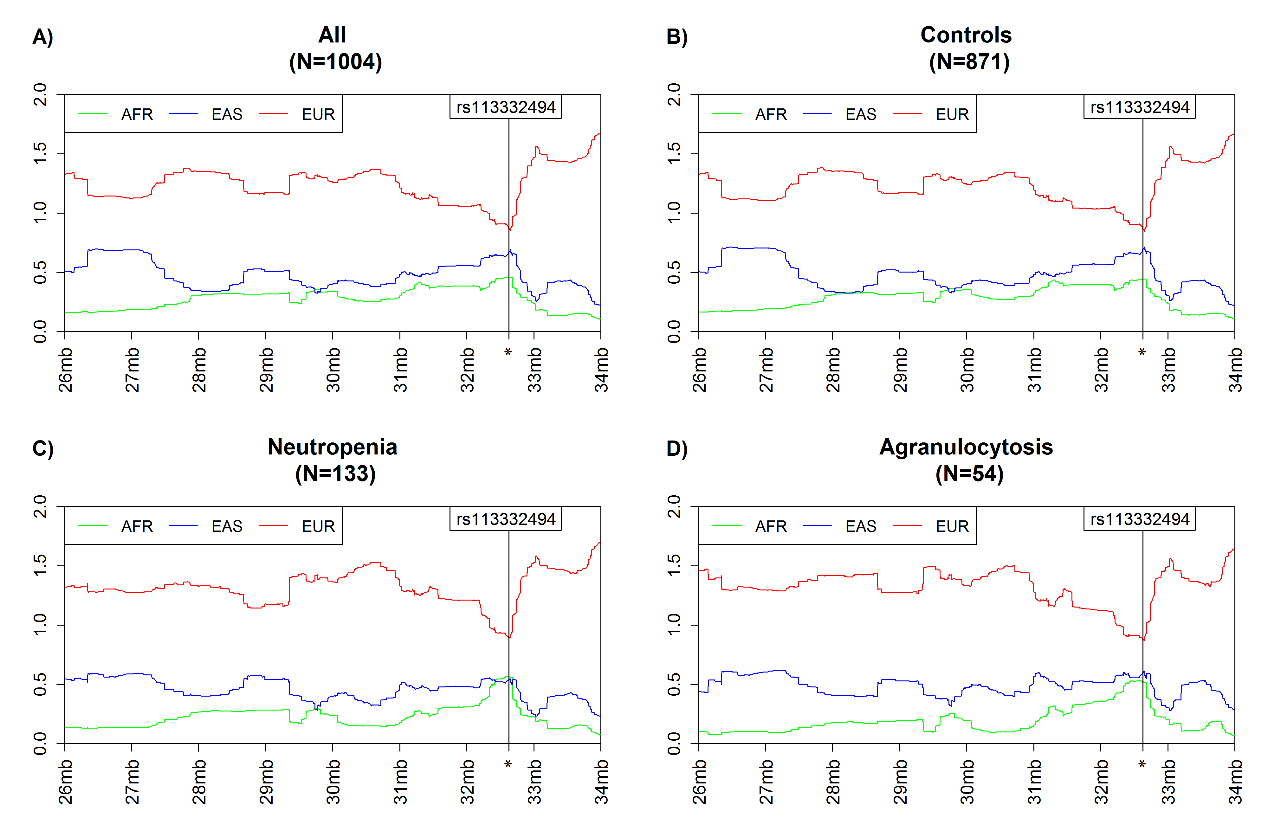


#### Supplementary Figure S4: Local ancestry estimates (defined on the basis of best guess genotypes with posterior probability > 0.7) estimated by ELAI using 1KG AFR, EAS and EUR super populations as reference. The x-axis and y-axis represent chromosomal position (hg19) and allele dosage estimates (green = AFR, blue = EAS, red = EUR). The vertical line labeled by an asterisk shows the position of marker rs113332494. Estimates are averaged across four groups of European ancestry: A) all individuals B) controls C) neutropenia cases D) agranulocytosis cases.


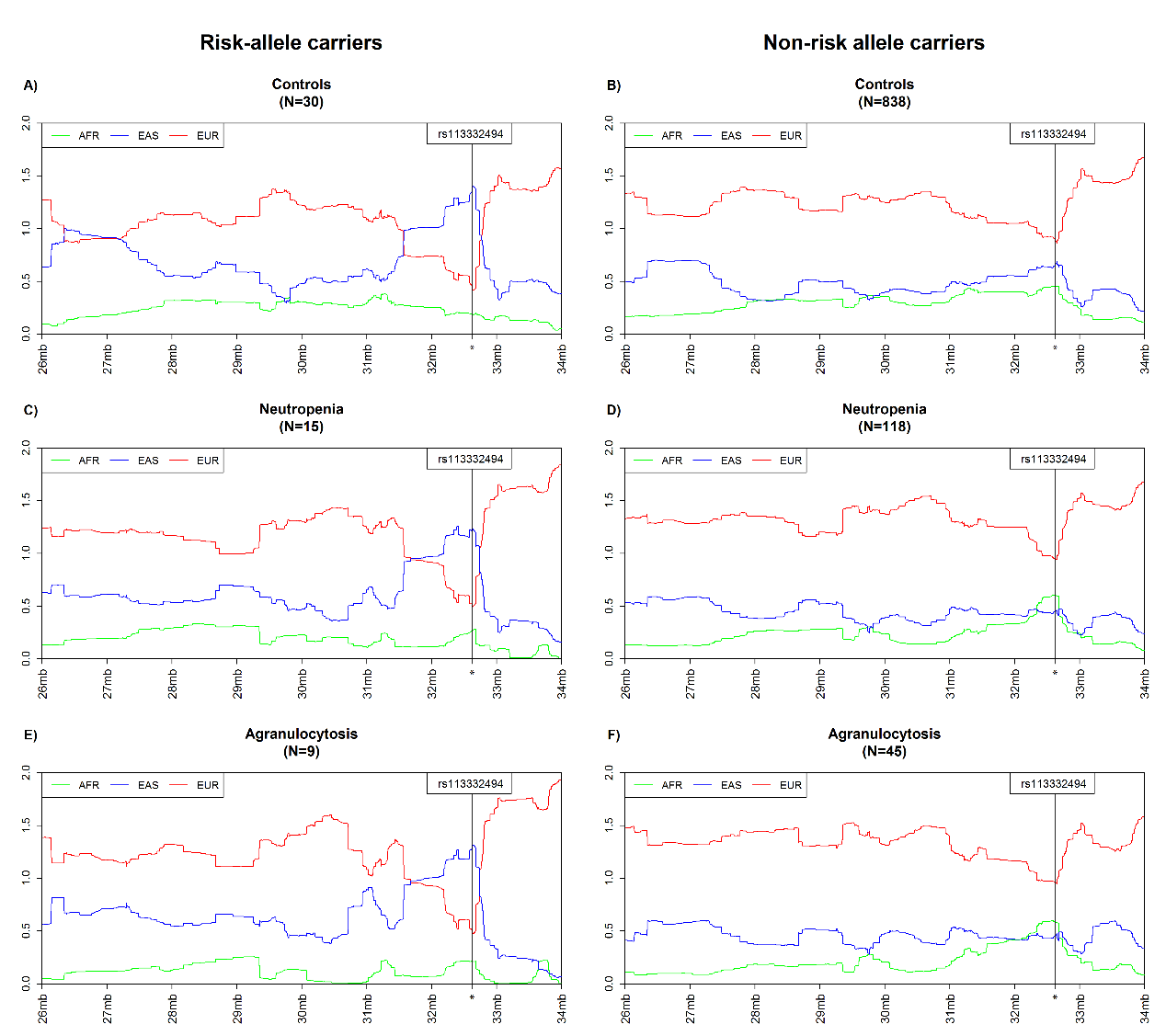


#### Supplementary Figure S5: Local ancestry of risk allele and non-risk allele carriers estimated by ELAI using 1KG AFR, EAS and EUR super populations as reference. The x-axis and y-axis represent chromosomal position (hg19) and allele dosage estimates for ancestral populations (green = AFR, blue = EAS, red = EUR) summing up to two. The vertical line labeled by an asterisk shows the position of marker rs113332494. Estimates are averaged across controls (A + B), neutropenia (C + D) and agranulocytosis (E + F) cases in individuals of European ancestry stratified by risk allele status (Left = Risk allele carriers, Right = Non-risk allele carriers). Risk allele status was defined using best guess genotypes with posterior probability > 0.7.


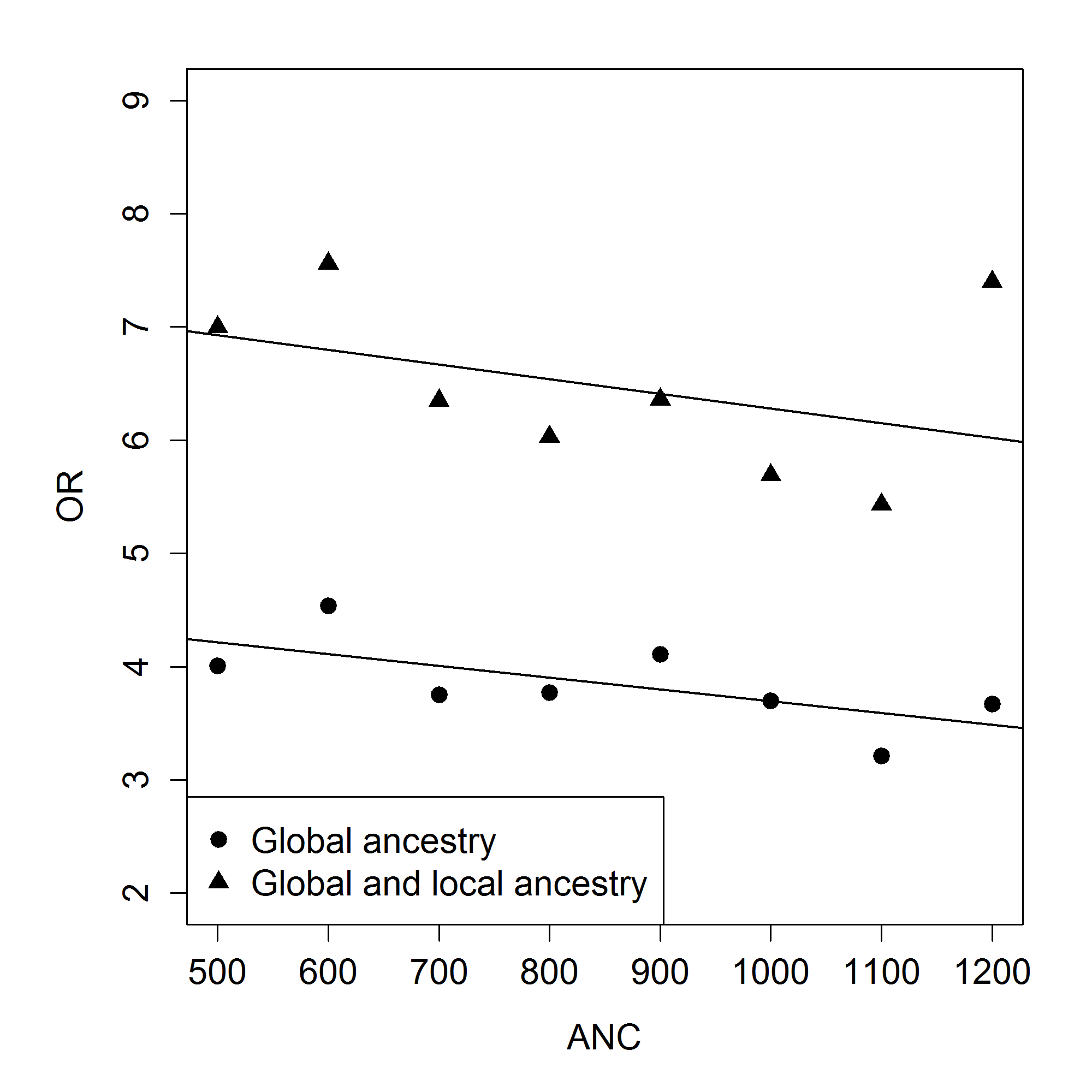


#### Supplementary Figure S6: Odds ratio estimates for varying ANC thresholds (500 - 1200) excluding all agranulocytosis cases. ANC gives the lowest absolute neutrophil count not included; e.g. ANC = 500 includes all cases with ANC = 501-1500. Odds ratios were determined in association analyses of European individuals corrected for global (principal components PC1 - PC7) and additionally for local ancestry (dosage estimates for ancestral populations AFR and EAS). Regression lines were determined by weighted least squares regression where the reciprocals of the standard errors were used as weights. Estimates are not given for ANC thresholds 1300 and 1400 as models could not be fitted due to low sample size and resulting multicollinearity issues.

1. AFR


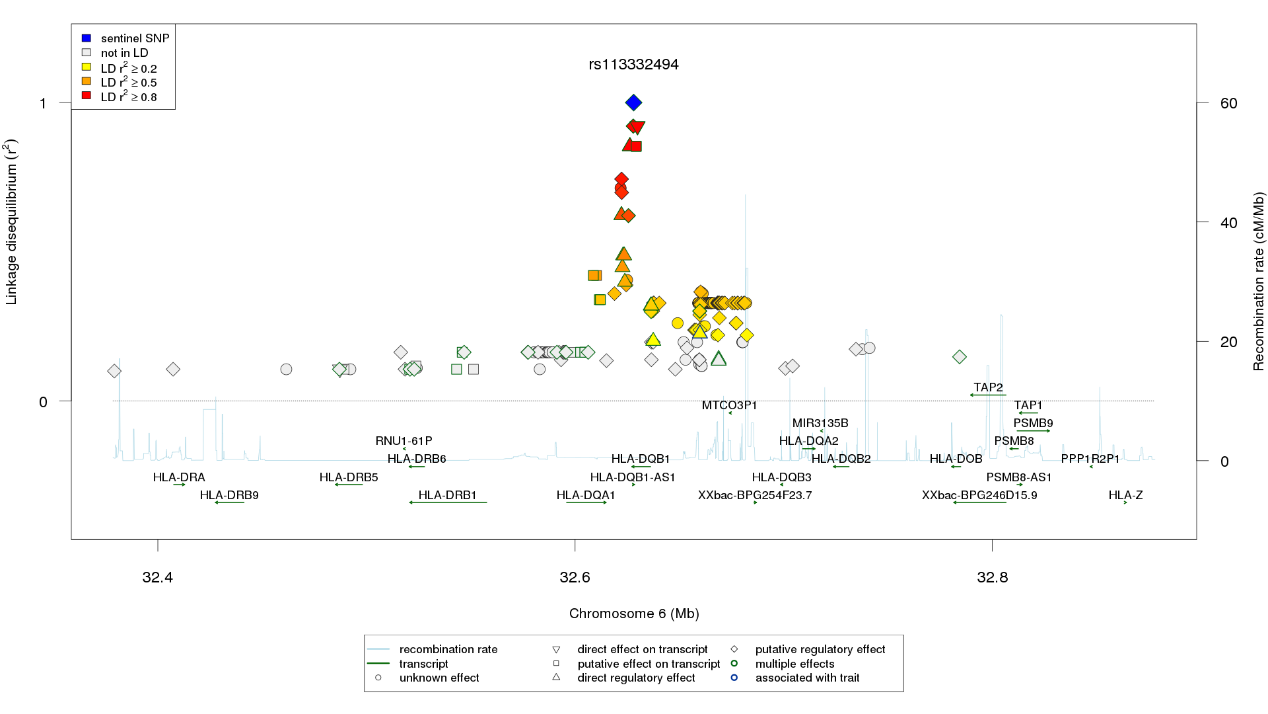


1. ASN


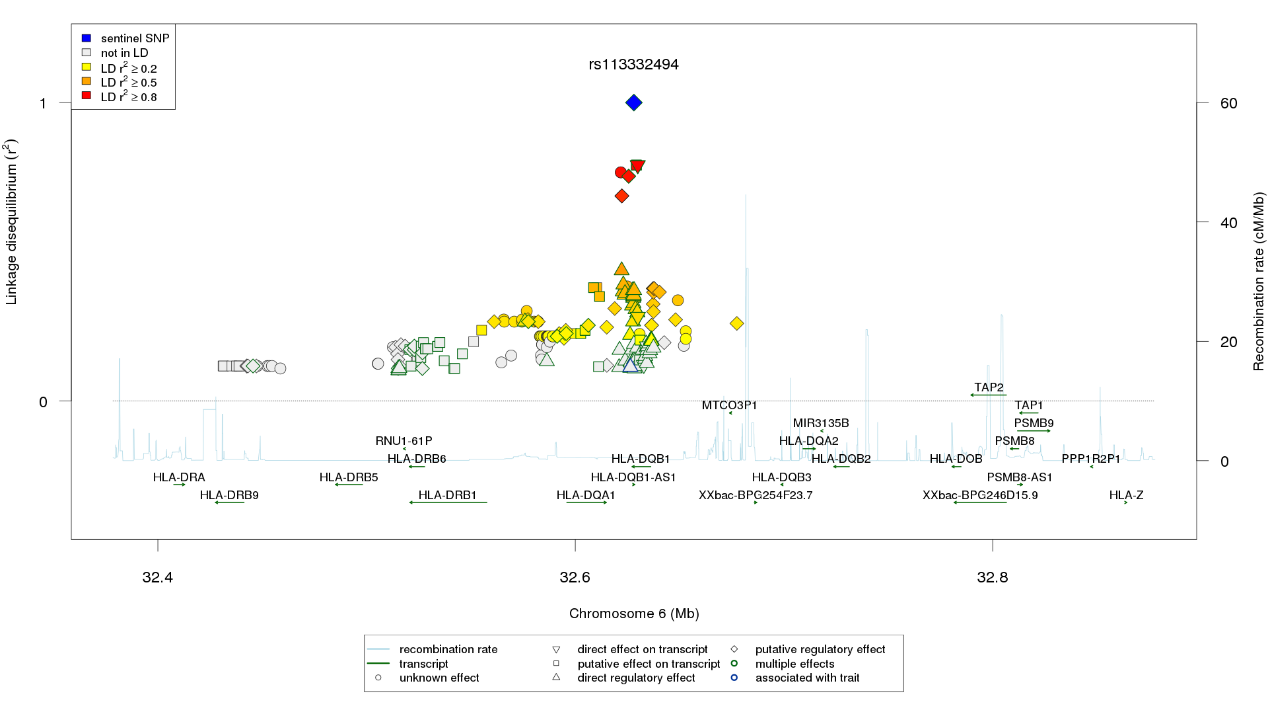


#### Supplementary Figure S7: Linkage disequilibrium plots of rs113332494 and surrounding markers coloured by LD patterns of AFR and ASN 1KG super-populations.

## References

1 Gaughran F *et al.* Improving physical health and reducing substance use in psychosis--randomised control trial (IMPACT RCT): study protocol for a cluster randomised controlled trial. *BMC Psychiatry* **13**, 263 (2013).

2 Stefansson H *et al.* Common variants conferring risk of schizophrenia. *Nature* **460**, 744–747 (2009).

3 First MB, Spitzer RL, Gibbon M, Williams JBW, Benjamin LS. *Structured Clinical Interview for DSM-IV Axis II Personality Disorders (SCID-II)*. American Psychiatric Press: Washington, DC, 1997.

4 First MB, Spitzer RL, Gibbon M, Williams JBW, Benjamin LS. *Structured Clinical Interview for DSM-IV-TR Axis I Disorders, Research Version, Patient Edition. (SCID-I/P)*. Biometrics Research, New York State Psychiatric Institute: New York, 2002.

5 Schulte PFJ, Cohen D, Bogers JPAM, van Dijk D, Bakker B. A Dutch guideline for the use of clozapine. *Aust. N. Z. J. Psychiatry* **44**, 1055–1056 (2010).

6 Rees E *et al.* Analysis of copy number variations at 15 schizophrenia-associated loci. *Br. J. Psychiatry J. Ment. Sci.* **204**, 108–114 (2014).

7 Schizophrenia Working Group of the Psychiatric Genomics Consortium. Biological insights from 108 schizophrenia-associated genetic loci. *Nature* **511**, 421–427 (2014).

8 Pardiñas AF *et al.* Common schizophrenia alleles are enriched in mutation-intolerant genes and in regions under strong background selection. *Nat. Genet.* **50**, 381–389 (2018).

9 Carroll LS *et al.* Mutation screening of the 3q29 microdeletion syndrome candidate genes DLG1 and PAK2 in schizophrenia. *Am. J. Med. Genet. Part B Neuropsychiatr. Genet. Off. Publ. Int. Soc. Psychiatr. Genet.* **156B**, 844–849 (2011).

10 Purcell S, Chang C. *PLINK 1.9 (https://www.cog-genomics.org/plink2)*. https://www.cog-genomics.org/plink2.

11 Chang CC *et al.* Second-generation PLINK: rising to the challenge of larger and richer datasets. *GigaScience* **4** (2015). doi:10.1186/s13742-015-0047-8.

12 Southam L *et al.* The effect of genome-wide association scan quality control on imputation outcome for common variants. *Eur. J. Hum. Genet.* **19**, 610–614 (2011).

13 Roshyara NR, Kirsten H, Horn K, Ahnert P, Scholz M. Impact of pre-imputation SNP-filtering on genotype imputation results. *BMC Genet.* **15** (2014). doi:10.1186/s12863-014-0088-5.

14 Howie B, Fuchsberger C, Stephens M, Marchini J, Abecasis GR. Fast and accurate genotype imputation in genome-wide association studies through pre-phasing. *Nat. Genet.* **44**, 955–959 (2012).

15 Howie B, Marchini J, Stephens M. Genotype imputation with thousands of genomes. *G3 Bethesda Md* **1**, 457–470 (2011).

16 Howie BN, Donnelly P, Marchini J. A flexible and accurate genotype imputation method for the next generation of genome-wide association studies. *PLoS Genet.* **5**, e1000529 (2009).

17 Delaneau O, Zagury J-F, Marchini J. Improved whole-chromosome phasing for disease and population genetic studies. *Nat. Methods* **10**, 5–6 (2012).

18 Price AL *et al.* Principal components analysis corrects for stratification in genome-wide association studies. *Nat. Genet.* **38**, 904–909 (2006).

19 Zhou H, Alexander D, Lange K. A quasi-Newton acceleration for high-dimensional optimization algorithms. *Stat. Comput.* **21**, 261–273 (2011).

20 McVean GA *et al.* An integrated map of genetic variation from 1,092 human genomes. *Nature* **491**, 56–65 (2012).

21 Legge SE *et al.* Genome-wide common and rare variant analysis provides novel insights into clozapine-associated neutropenia. *Mol. Psychiatry* **22**, 1502–1508 (2017).

22 Goldstein JI *et al.* Clozapine-induced agranulocytosis is associated with rare HLA-DQB1 and HLA-B alleles. *Nat. Commun.* **5**, 4757 (2014).
